# Supplementary figures and images for: Effects of replication domains on genome-wide UV-induced DNA damage and repair
Source: PLoS Genet. 2022 Sep 26;18(9):e1010426. doi: 10.1371/journal.pgen.1010426 (PMC9536635; doi:10.1371/journal.pgen.1010426)

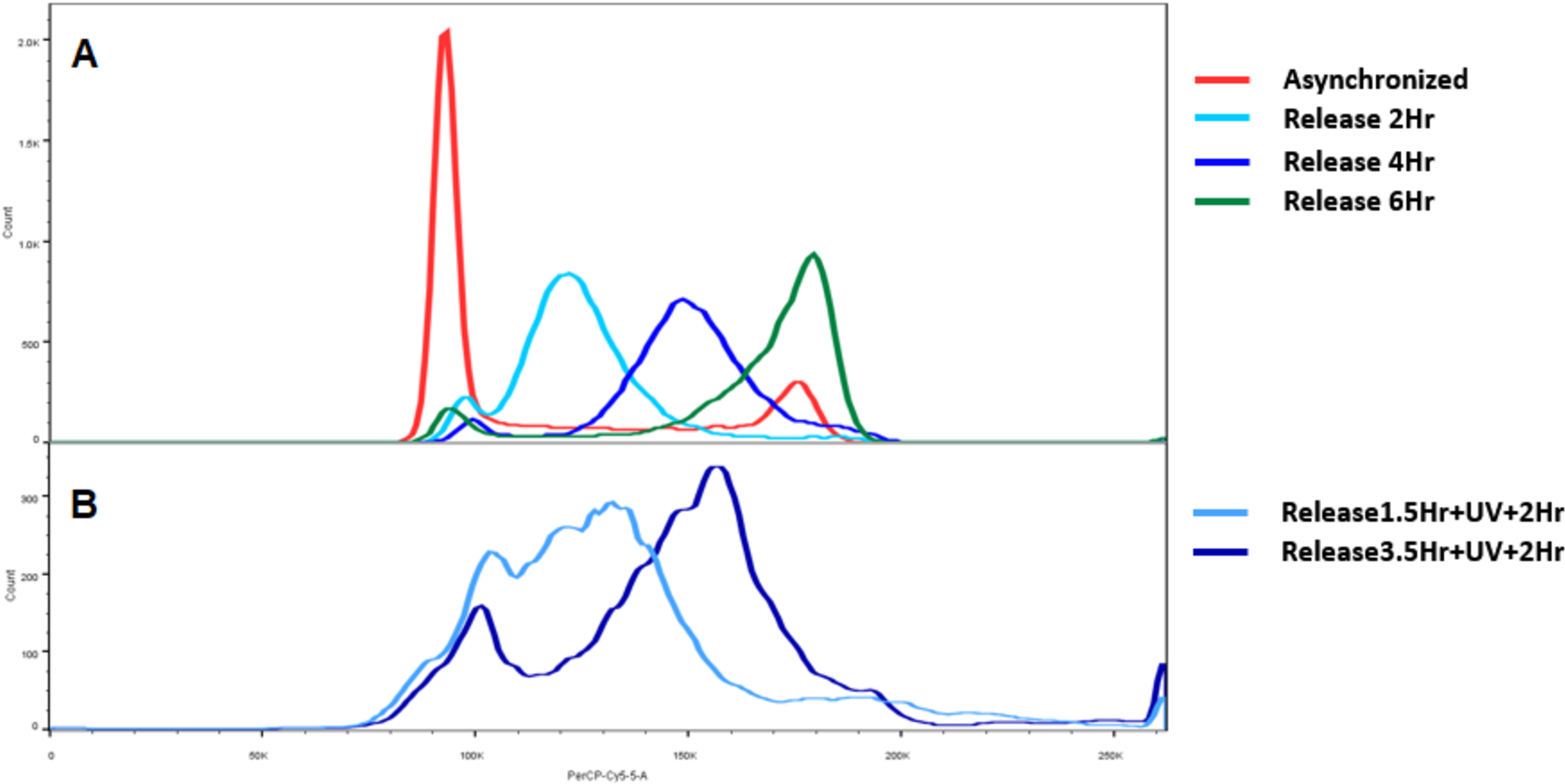

Supplement: S1 Fig — (A), the distribution of DNA contents of asynchronized HeLa cells (red) was merged with double-thymidine synchronized cells which were released to S phase for 2h (cyan), 4h (blue) and 6h (green). (B), DNA contents of synchronized HeLa cells treated by UVC. Cells were released to S phase for 1.5h (light blue) or 3.5h (dark blue), followed by UV irradiation and 2h-incubation before flow cytometry analysis. (TIF) [file pgen.1010426.s001.tif]

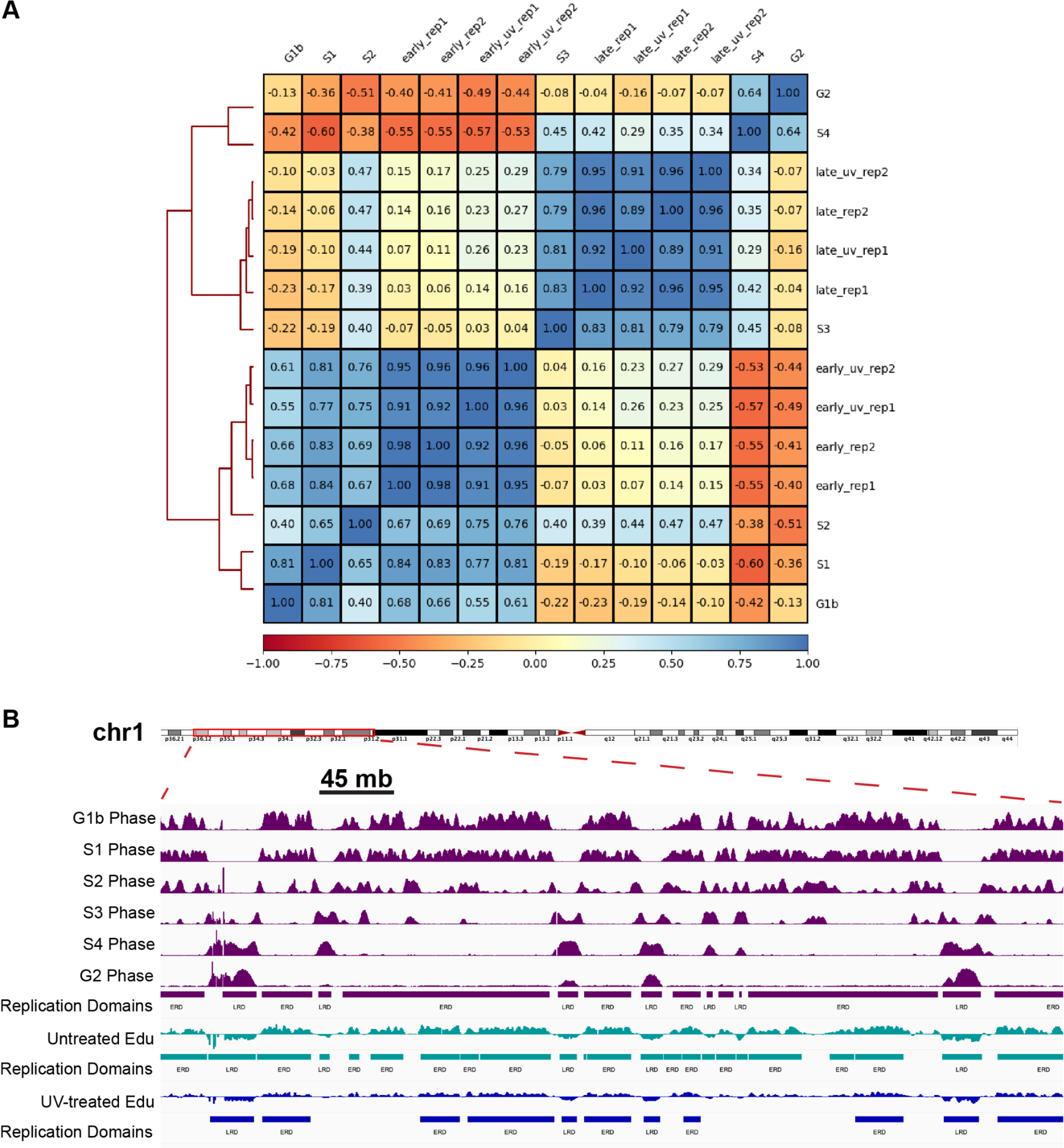

Supplement: S2 Fig — (A) Heatmap showing the pairwise comparison of spearman correlation coefficient between UV-treated/untreated early and late S phased EdU-seq samples (2 replicates for each) and G1b, S (1–4), G2 phases of public HeLa-S3 Repli-seq data. (B) Screenshot of IGV tracks. Tracks 1–6: Repli-seq signals of G1b to G2 phases (purple); track 7: the replication domains determined by Repli-seq (purple); track 8: early (top)/late (bottom) S phase signals of untreated EdU-seq; track 9: the replication domains determined by untreated EdU-seq; track 10: early (top)/late (bottom) S phase signals of UV-treated EdU-seq; track 11: the replication domains determined by UV-treated EdU-seq. (TIF) [file pgen.1010426.s002.tif]

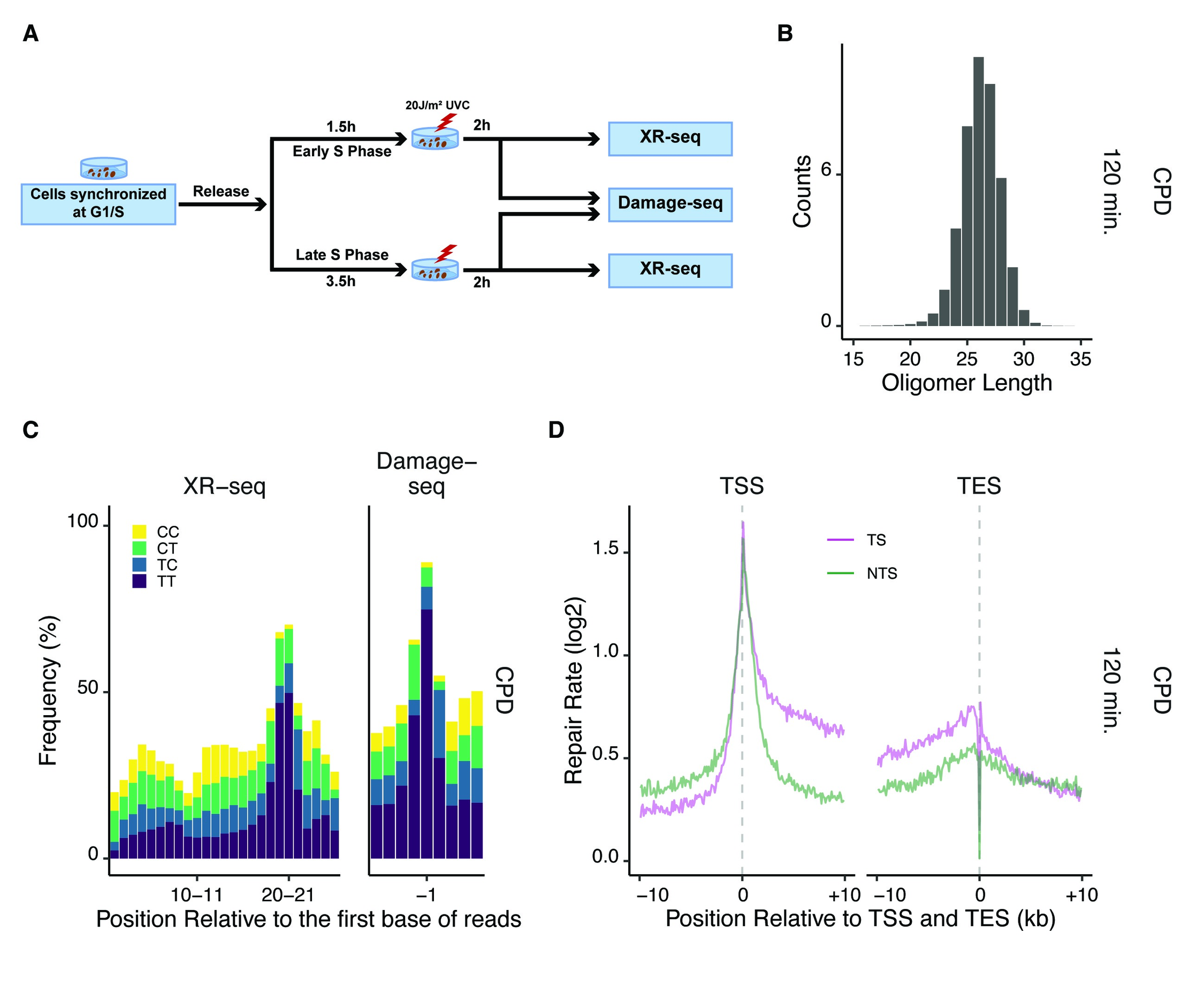

Supplement: S3 Fig — Same as Fig 1 except that synchronized HeLa cells were irradiated at 1.5 h and 3.5 h after release and collected at 2 h after UVC irradiation. (B-C) Replicate A is shown. (D) Replicate A and B are combined. (TIF) [file pgen.1010426.s003.tif]

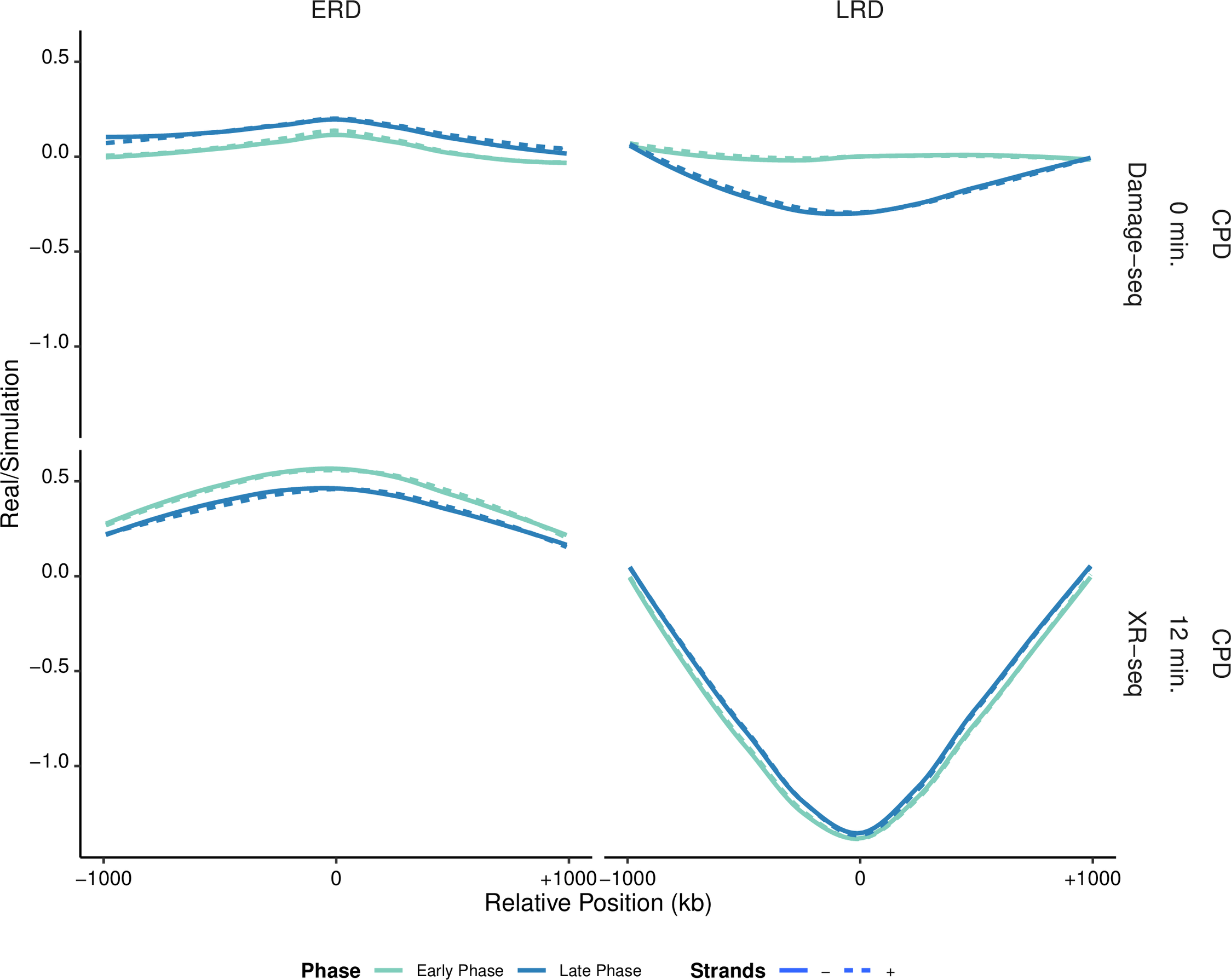

Supplement: S4 Fig — Replicate A and B are combined. (TIF) [file pgen.1010426.s004.tif]

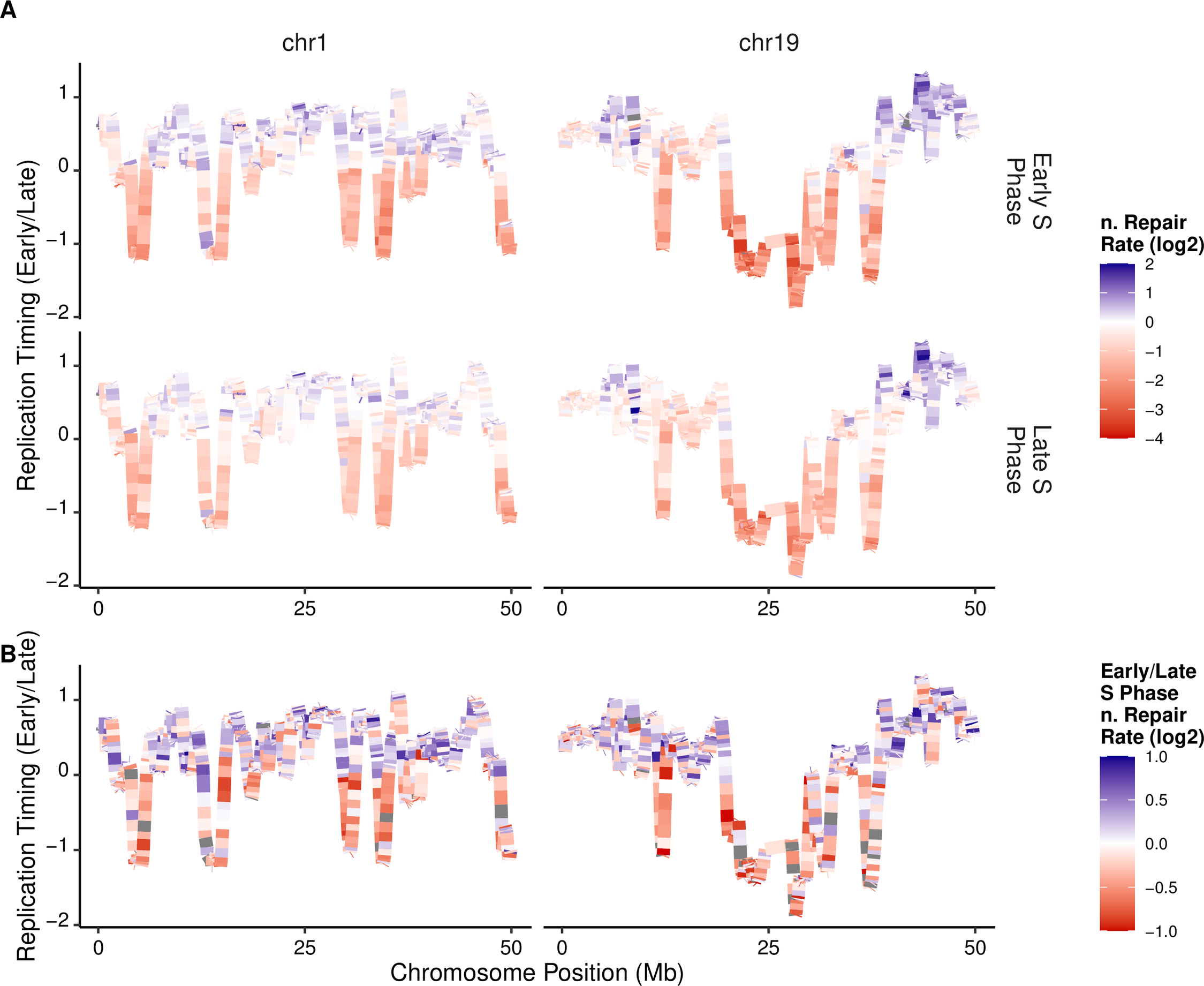

Supplement: S5 Fig — Replication timing profiles are shown for chromosome 1 and 19 until 50 Mb. Profiles are colored by the local ratio of (A) normalized repair rate and (B) Early/Late S phase normalized repair rate of CPD 12 minutes samples. (TIF) [file pgen.1010426.s005.tif]

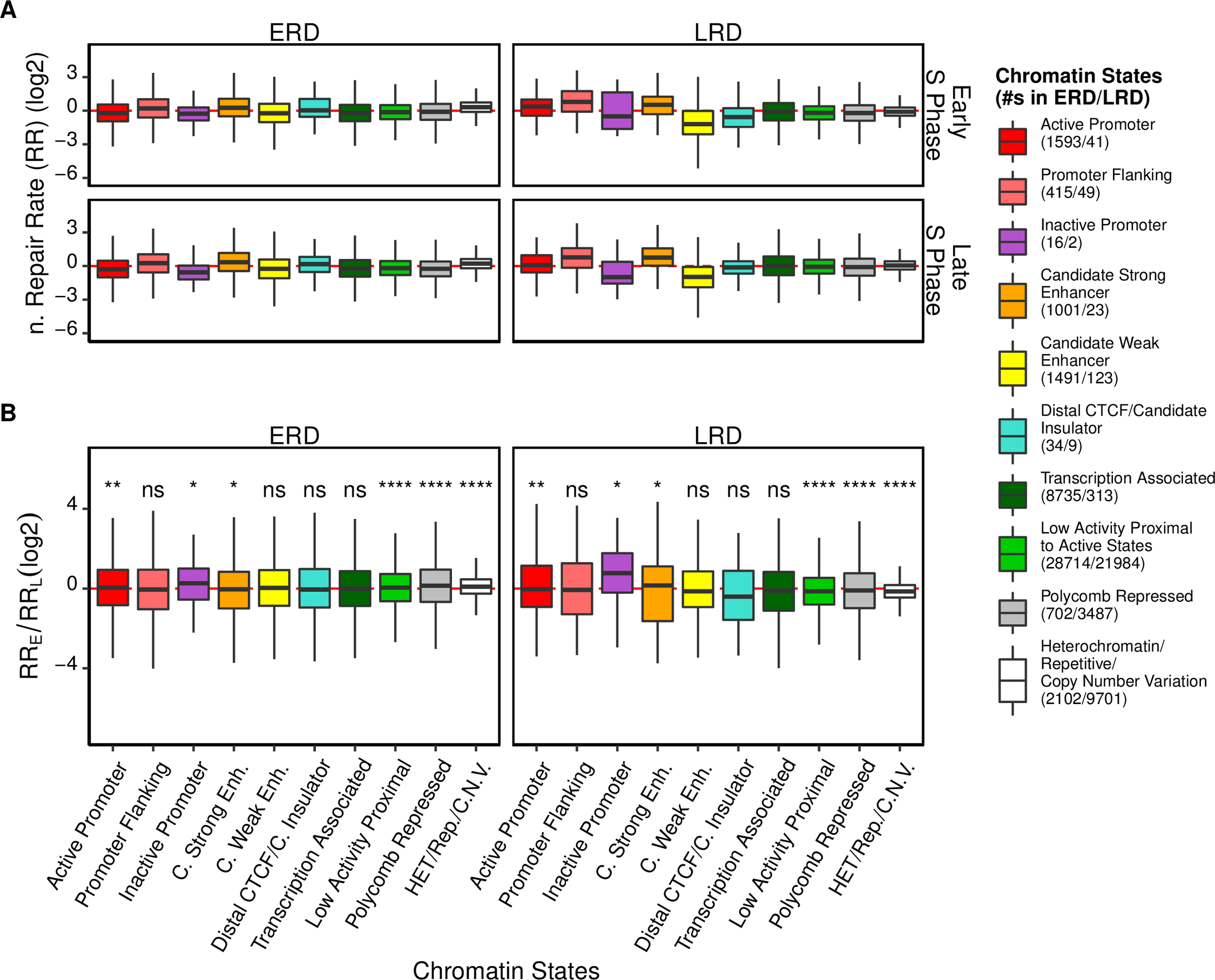

Supplement: S6 Fig — Same as Fig 3 except that the repair of (6–4)PPs was presented. Replicate A and B are combined. (TIF) [file pgen.1010426.s006.tif]

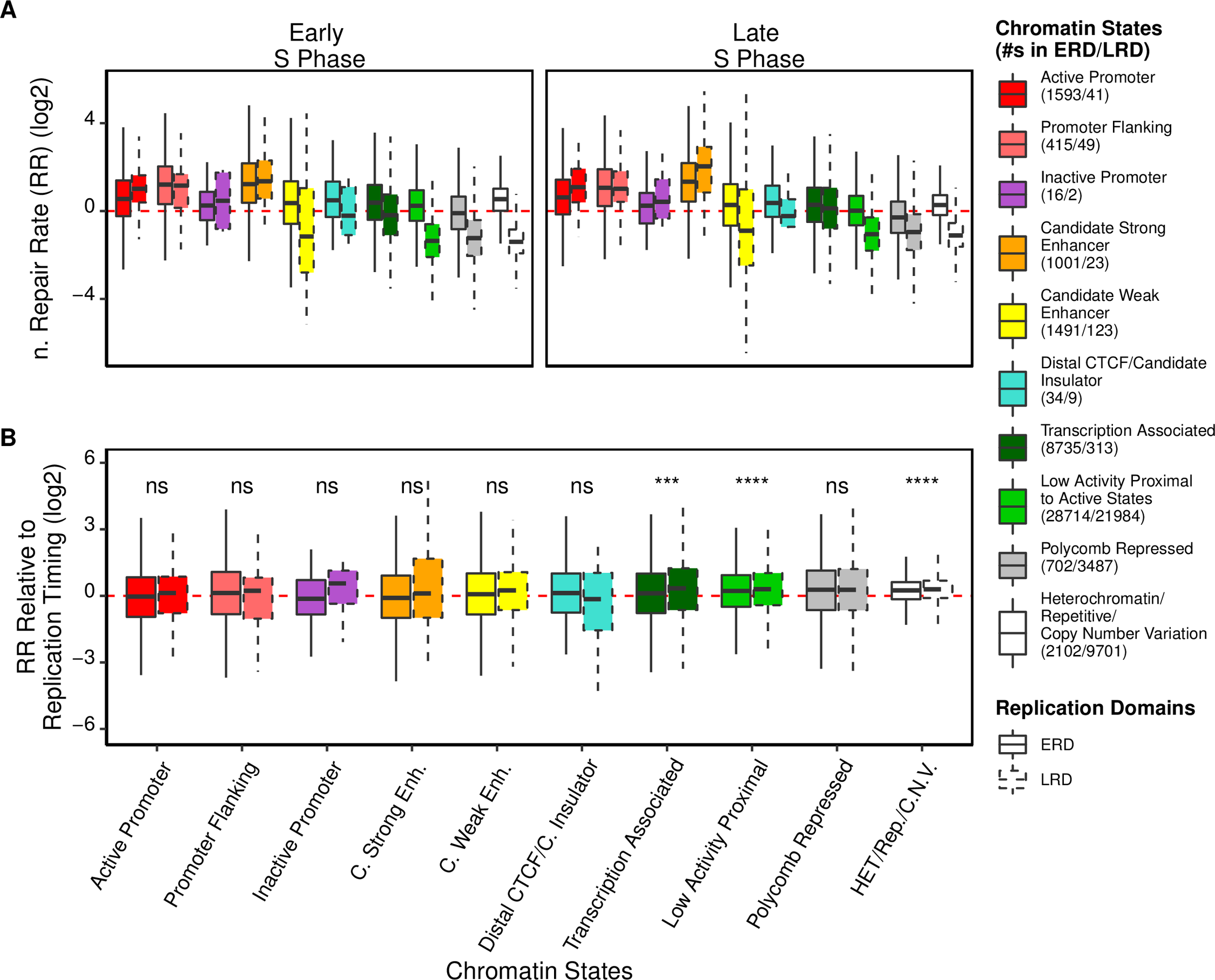

Supplement: S7 Fig — (A) Boxplot of normalized repair rates of CPDs at 12 minutes after UV at early and late S phases for each chromatin state. (B) Relative difference of early S phase and late S phase repair rates ([log2(RREarly S Phase/RRLate S Phase)] used for ERDs; [log2(RRLate S Phase/RREarly S Phase)] used for LRDs) for each chromatin state. The wilcoxon test was used to assess the significance of differences between ERDs and LRDs. Straight lines were ERDs, and dashed lines were LRDs. Replicate A and B are combined. (TIF) [file pgen.1010426.s007.tif]

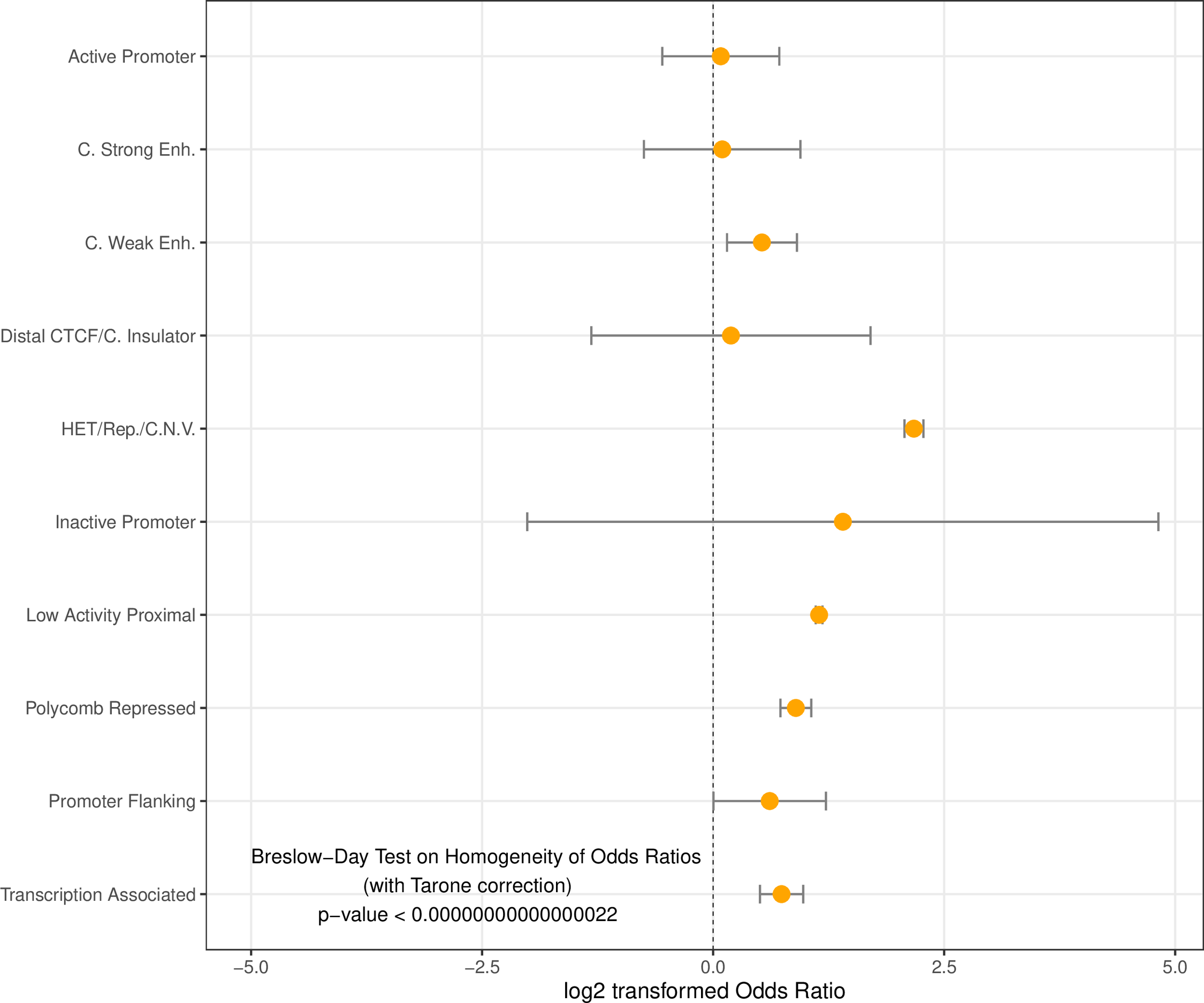

Supplement: S8 Fig — Chromatin states were categorized as being better repaired in early S phase or late S phase. Then, the association of ERDs (relative to LRDs) and odds of having better repair in early S phase than late S phase was calculated for each chromatin states. Horizontal lines show the lower and upper confidence intervals of odds ratios and Breslow-Day test (with Tarone correction) was used to test homogeneity of odds ratios across the chromatin states. (TIF) [file pgen.1010426.s008.tif]

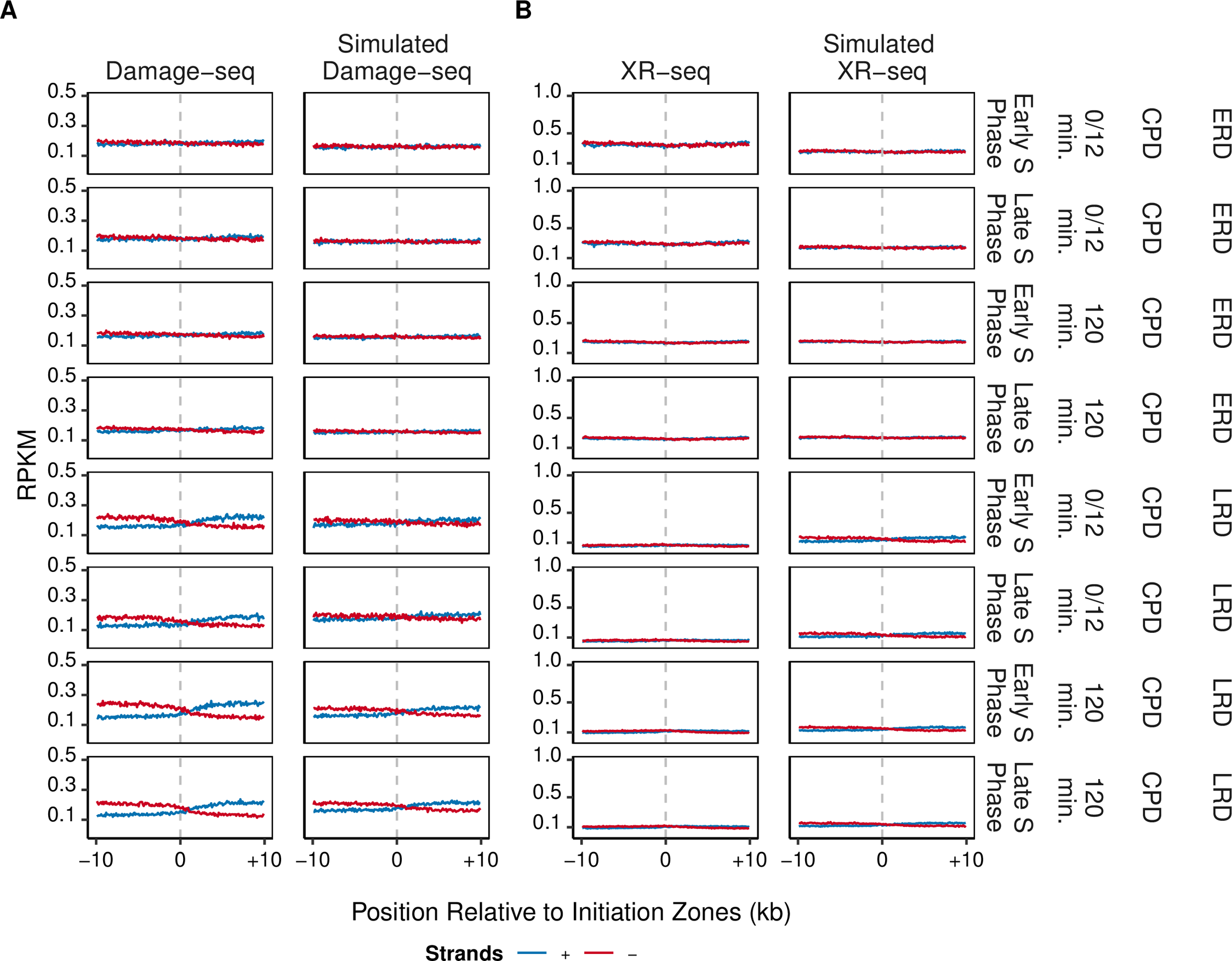

Supplement: S9 Fig — (A) Damage and (B) repair profiles of real and simulated reads around initiation zones for CPDs at 0 minutes (for damage), 12 minutes (for repair), and 2 hours (for both damage and repair). Replicate A and B are combined. (TIF) [file pgen.1010426.s009.tif]

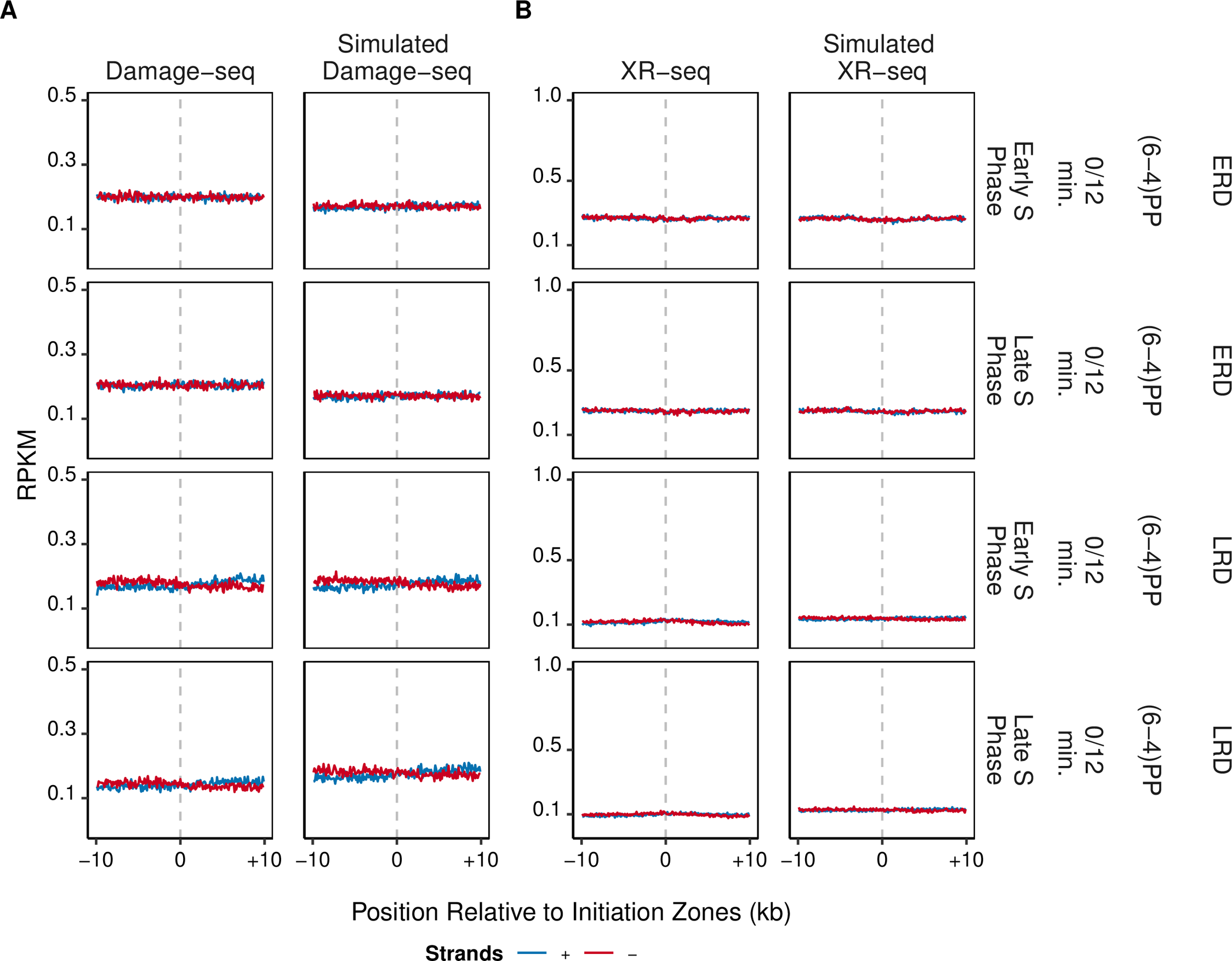

Supplement: S10 Fig — (A) Damage and (B) repair profiles of real and simulated reads around initiation zones for (6–4)PPs at 0 minutes and 12 minutes, respectively. Replicate A and B are combined. (TIF) [file pgen.1010426.s010.tif]

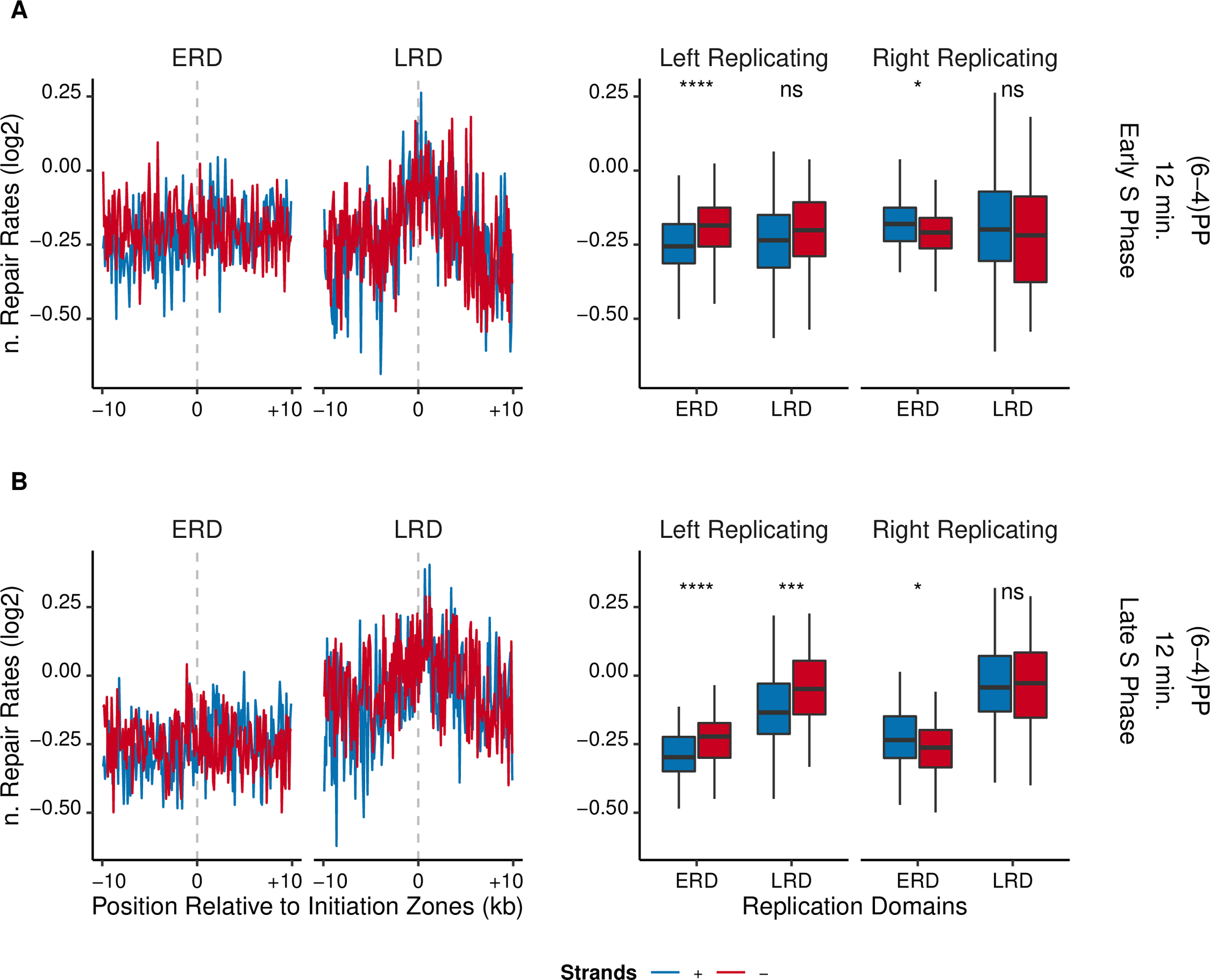

Supplement: S11 Fig — (Left) (6–4)PP normalized repair rates [log2 (XR-seqreal/simulation/Damage-seqreal/simulation)] at 12 minutes in early (A) and late (B) S phases around initiation zones that were separated into corresponding replication domains. (Right) The boxplot of windows in left replicating and right replicating directions are shown separately for plus and minus strands. Paired wilcoxon test was used to assess the significance of differences between the strands. Replicate A and B are combined. (TIF) [file pgen.1010426.s011.tif]

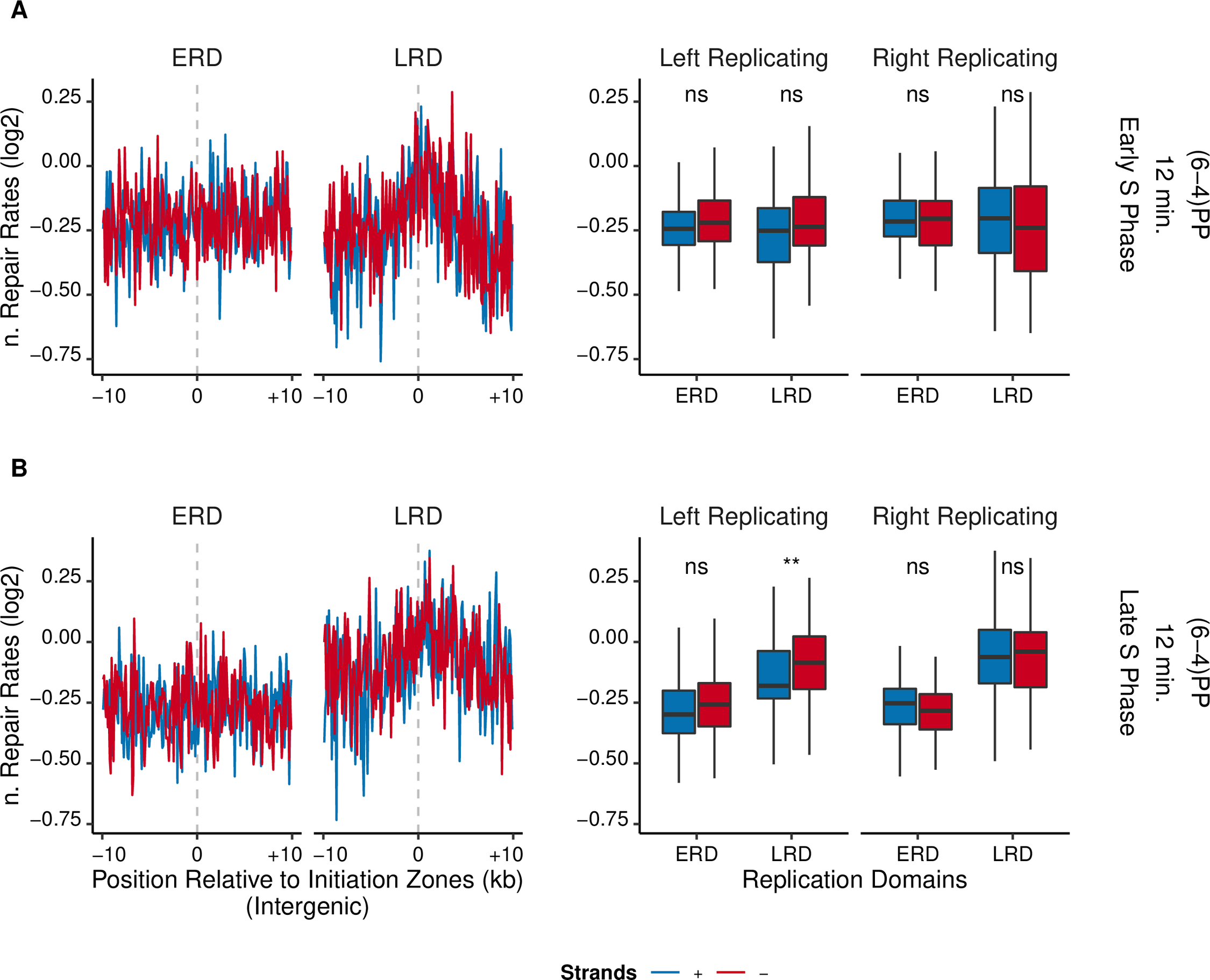

Supplement: S12 Fig — Similar as S11 Fig except that damage and repair signals in the annotated transcribed regions were discarded before analysis. Replicate A and B are combined. (TIF) [file pgen.1010426.s012.tif]

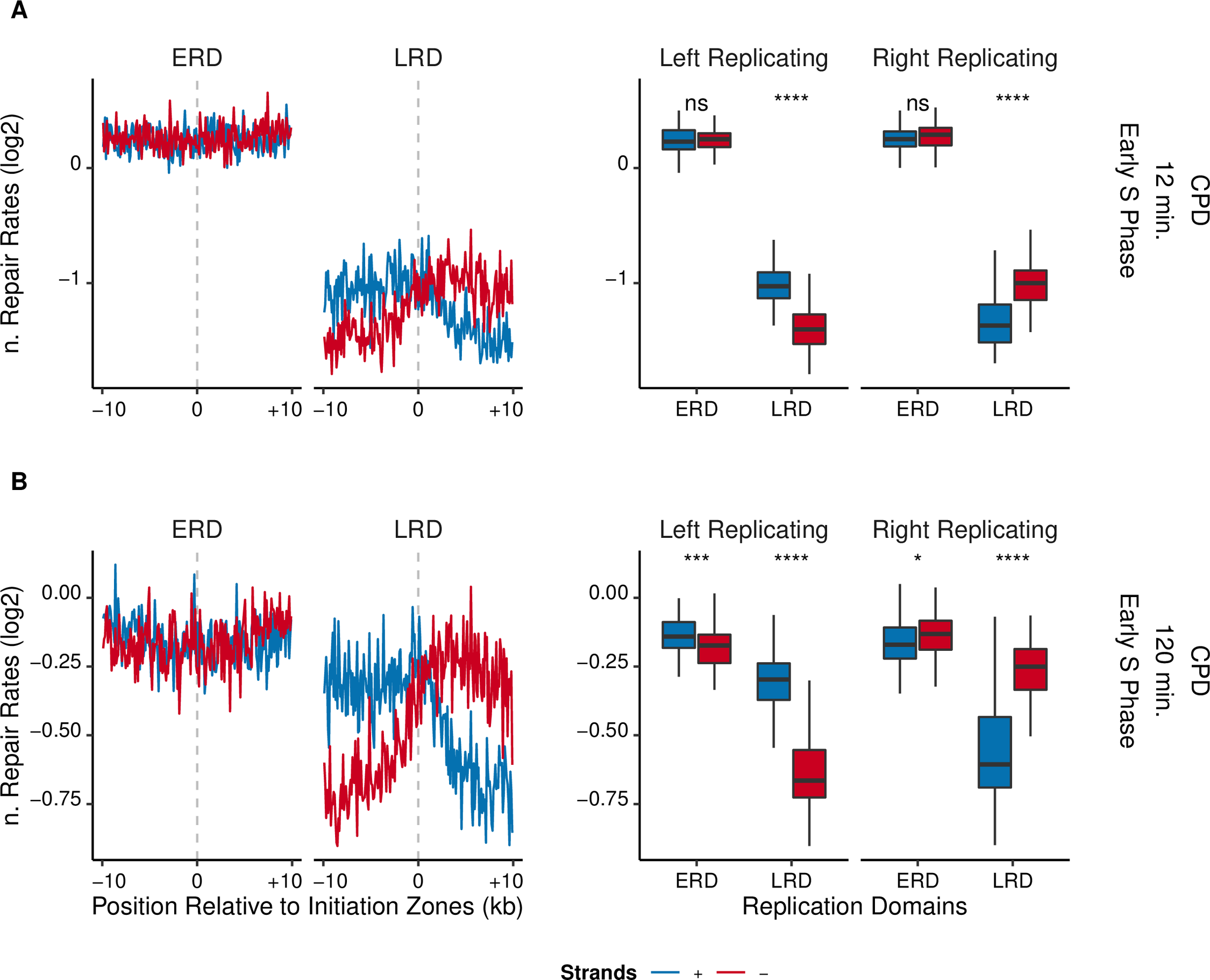

Supplement: S13 Fig — Same as Fig 4C and 4D except that cells were synchronized to the early S phase. (TIF) [file pgen.1010426.s013.tif]

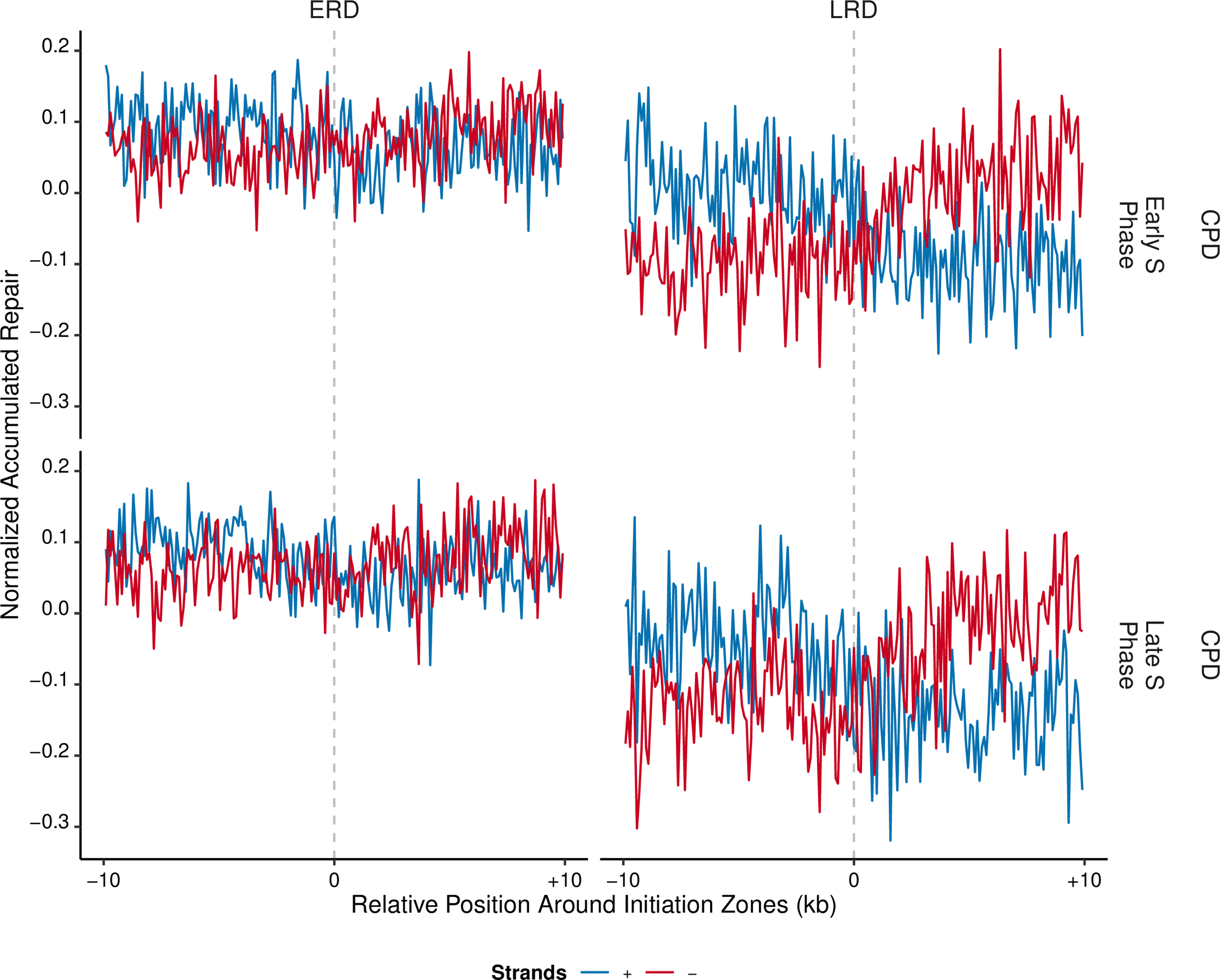

Supplement: S14 Fig — Normalized accumulated repair [(Damage-seq0h − Damage-seq2h) / Damage-seq0h] calculated for CPD samples at early and late S phases for ERDs and LRDs. (TIF) [file pgen.1010426.s014.tif]

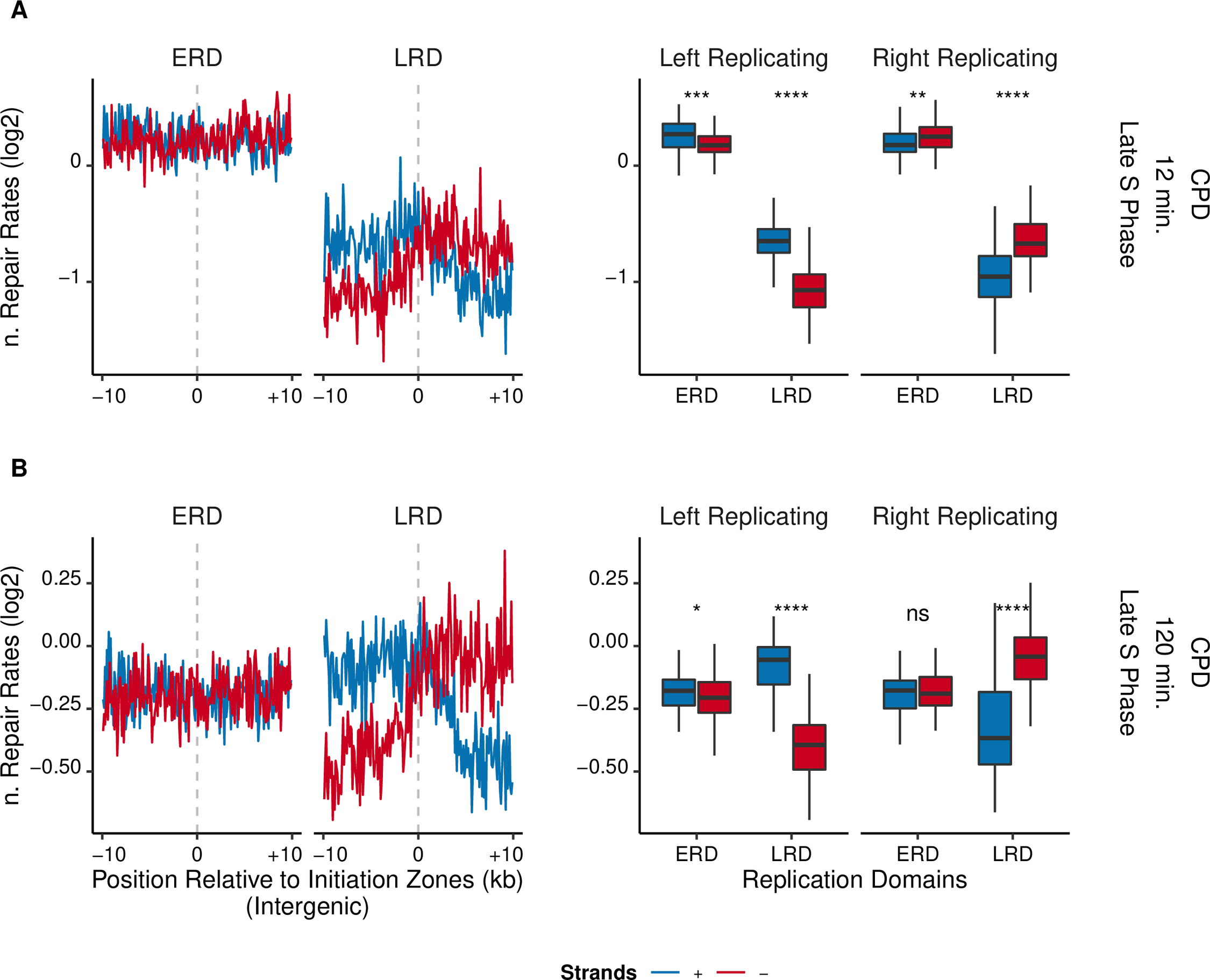

Supplement: S15 Fig — Similar as Fig 4C and 4D except that damage and repair signals in the annotated transcribed regions were discarded before analysis. Replicate A and B are combined. (TIF) [file pgen.1010426.s015.tif]

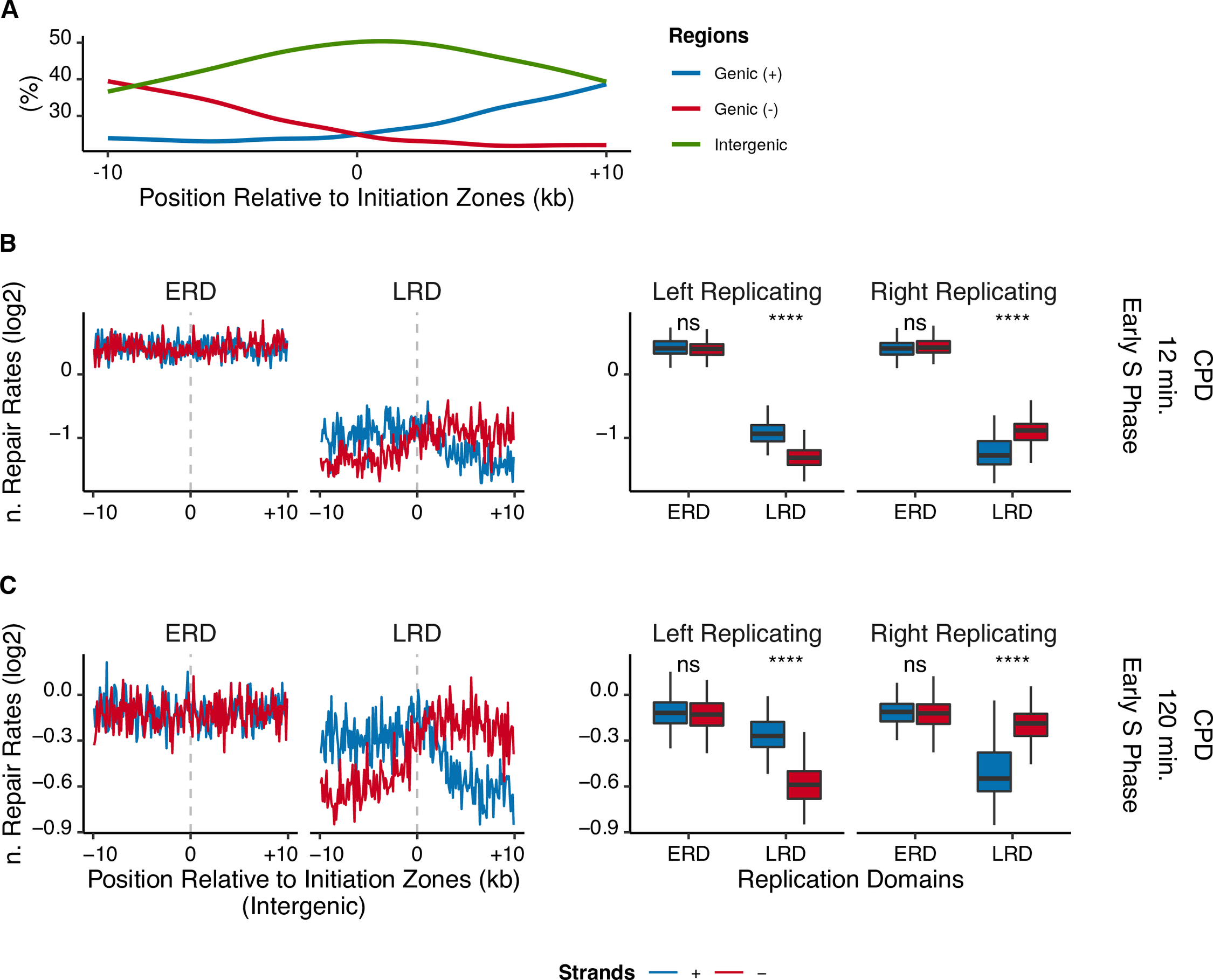

Supplement: S16 Fig — (A) Distribution of genic and intergenic regions around initiation zones. The blue line indicates genes located on plus strands, the red line indicates genes located on minus strands, and the green line indicates intergenic regions. (B-C) Same as S15 Fig except that cells were synchronized to the early S phase. Replicate A and B are combined. (TIF) [file pgen.1010426.s016.tif]

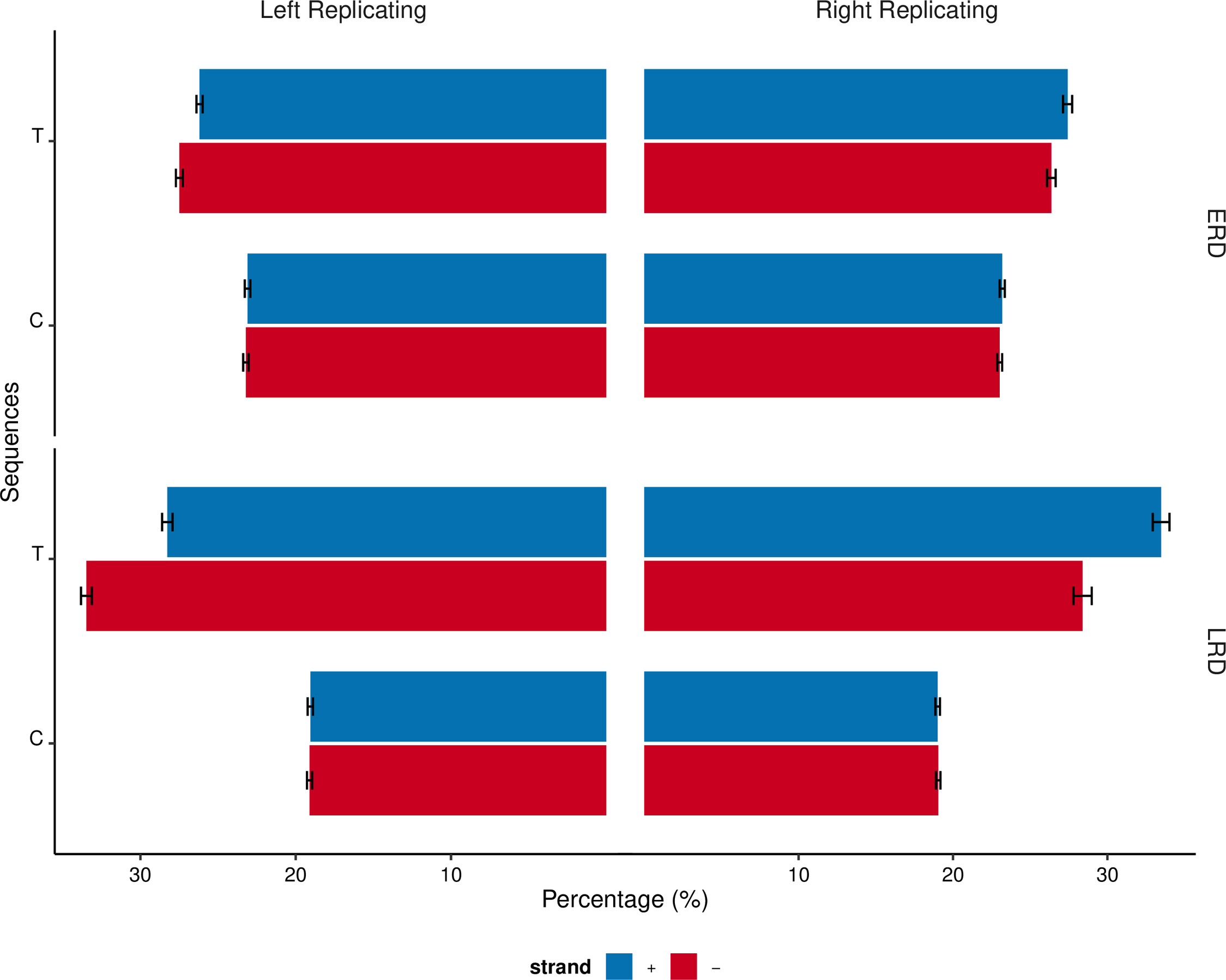

Supplement: S17 Fig — The percentage of each nucleotide at initiation zones in ERDs (Top) and LRDs (Bottom) was calculated separately for plus (blue) and minus (red) strands. Only a single sequence of a complementary pair was used. Sequences ordered based on the percentage differences between strands (top sequence having the highest difference while the bottom has the least). (TIF) [file pgen.1010426.s017.tif]

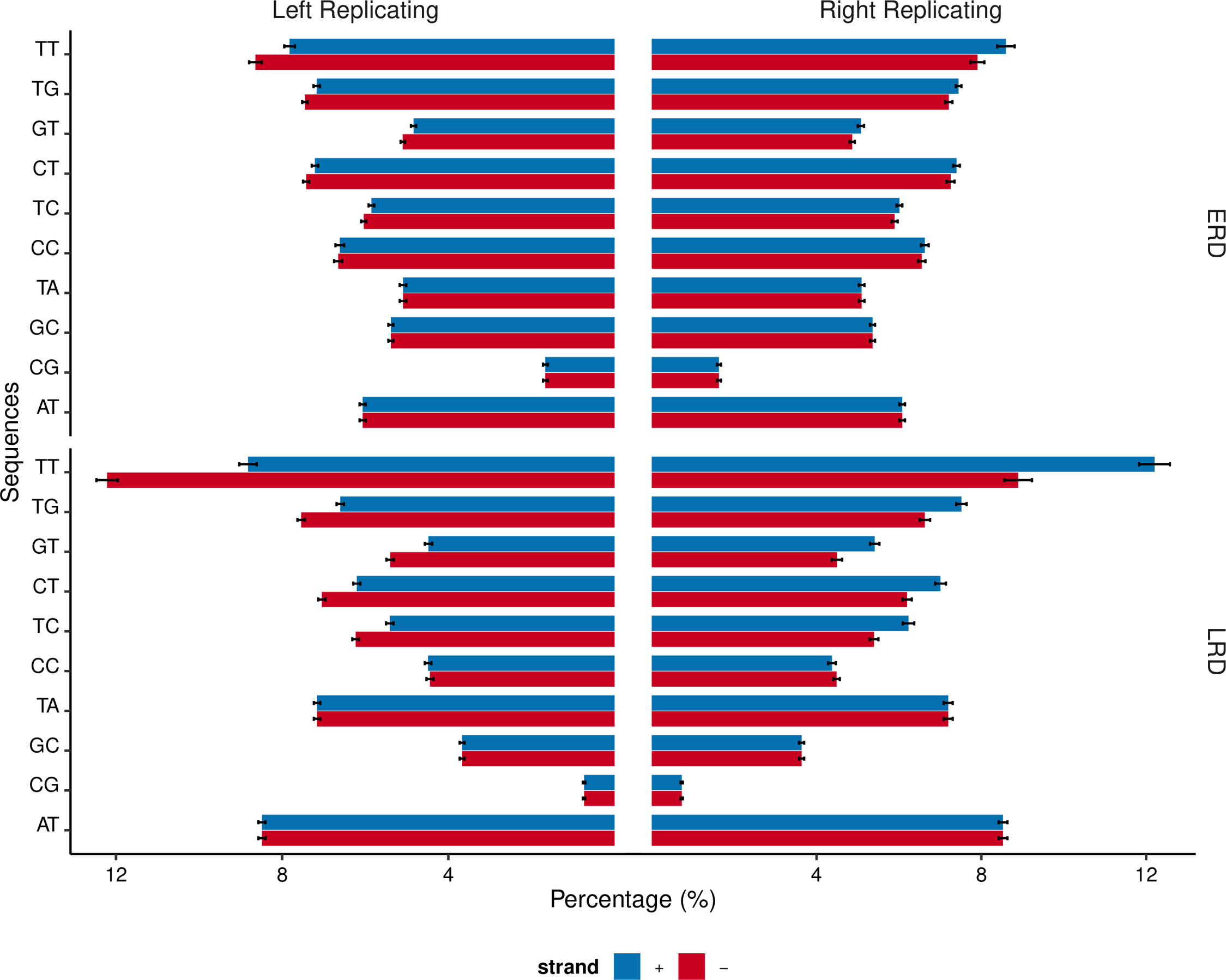

Supplement: S18 Fig — Similar to the S17 Fig except dinucleotides used instead of mononucleotides. (TIF) [file pgen.1010426.s018.tif]

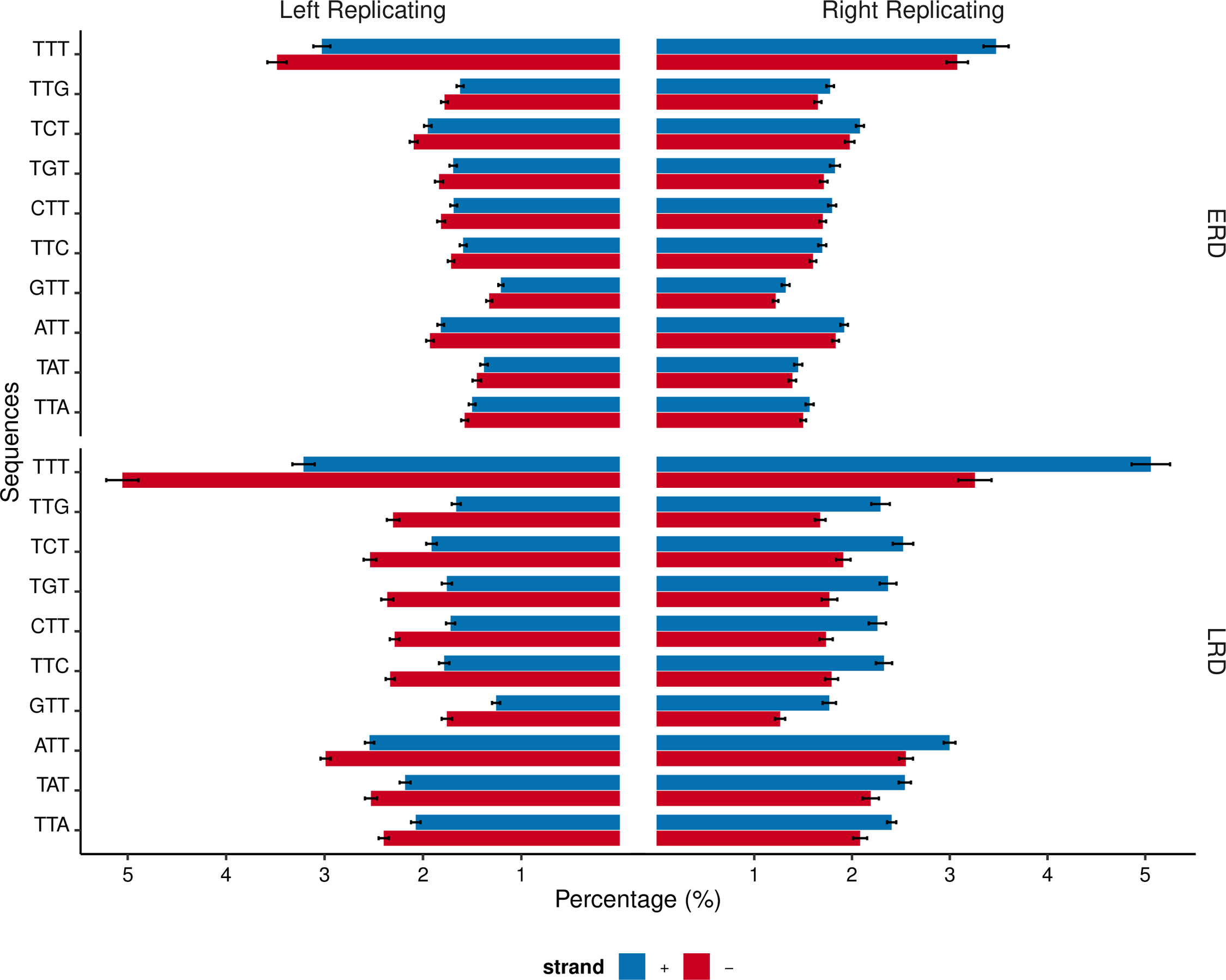

Supplement: S19 Fig — Similar to the S17 Fig except trinucleotides used instead of mononucleotides. Ten sequences with the highest difference were shown. (TIF) [file pgen.1010426.s019.tif]

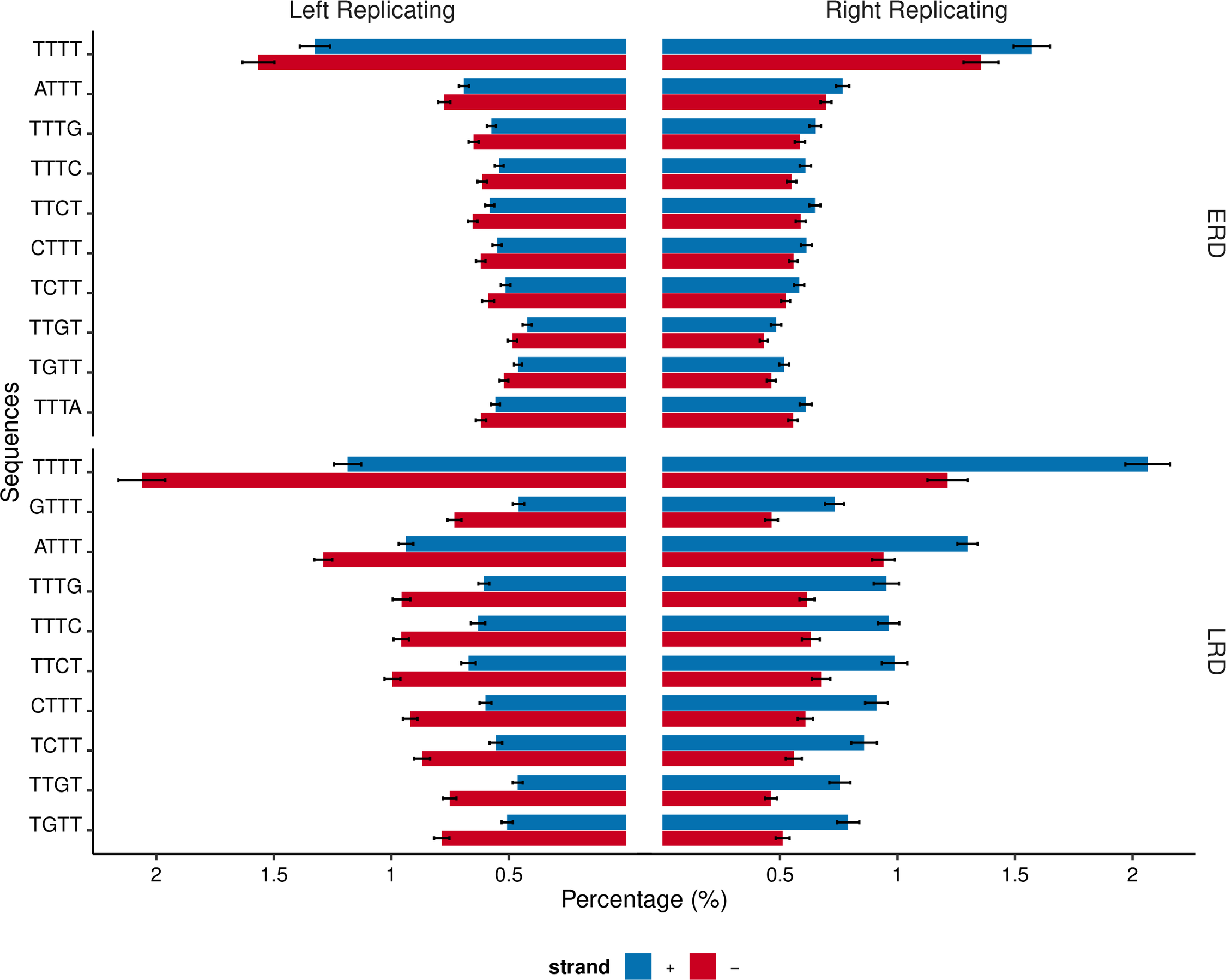

Supplement: S20 Fig — Similar to the S17 Fig except quadnucleotides used instead of mononucleotides. Ten sequences with the highest difference were shown. (TIF) [file pgen.1010426.s020.tif]

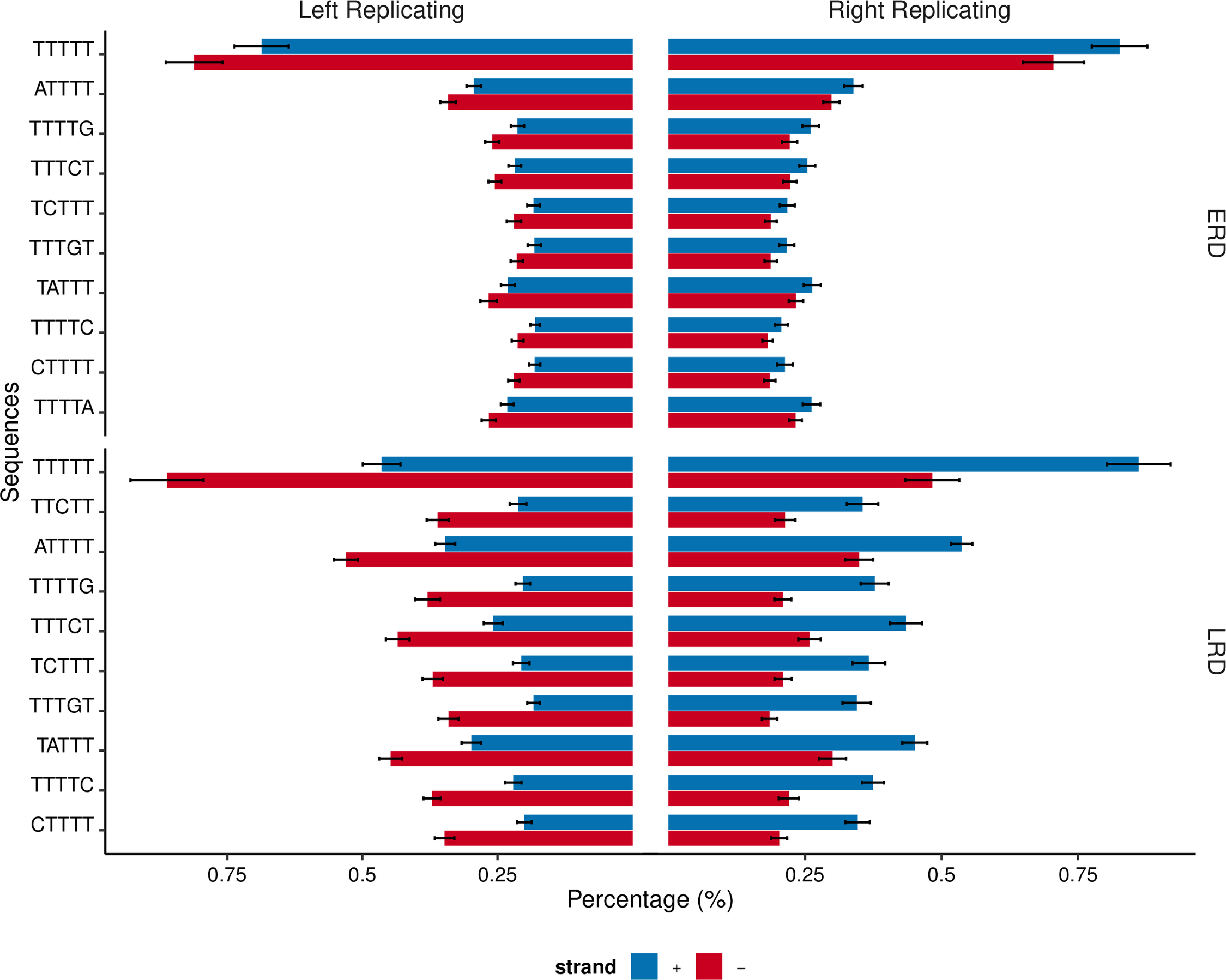

Supplement: S21 Fig — Similar to the S17 Fig except pentanucleotides used instead of mononucleotides. Ten sequences with the highest difference were shown. (TIF) [file pgen.1010426.s021.tif]

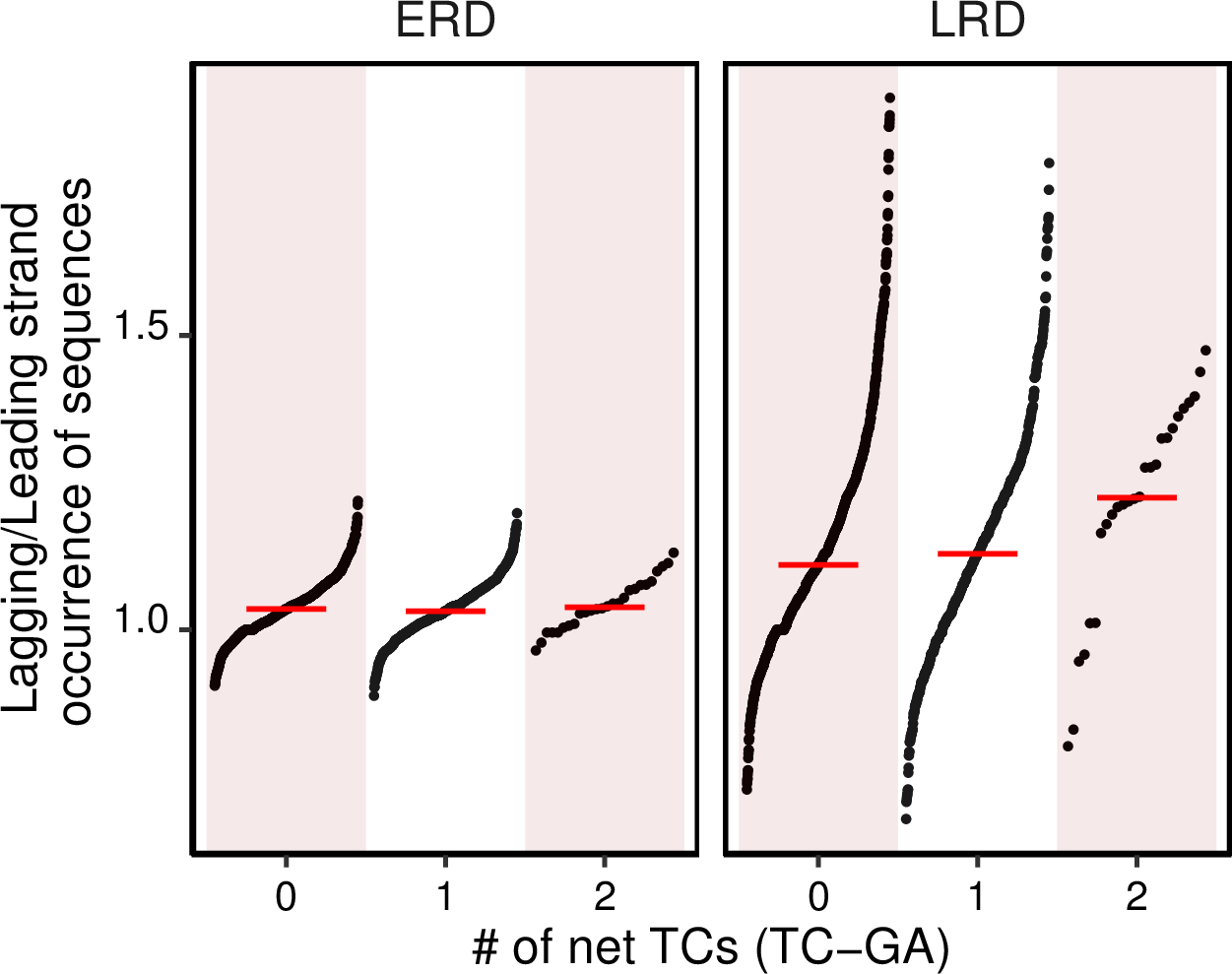

Supplement: S22 Fig — Same as Fig 4E except net TCs were calculated instead of net Ts. (TIF) [file pgen.1010426.s022.tif]

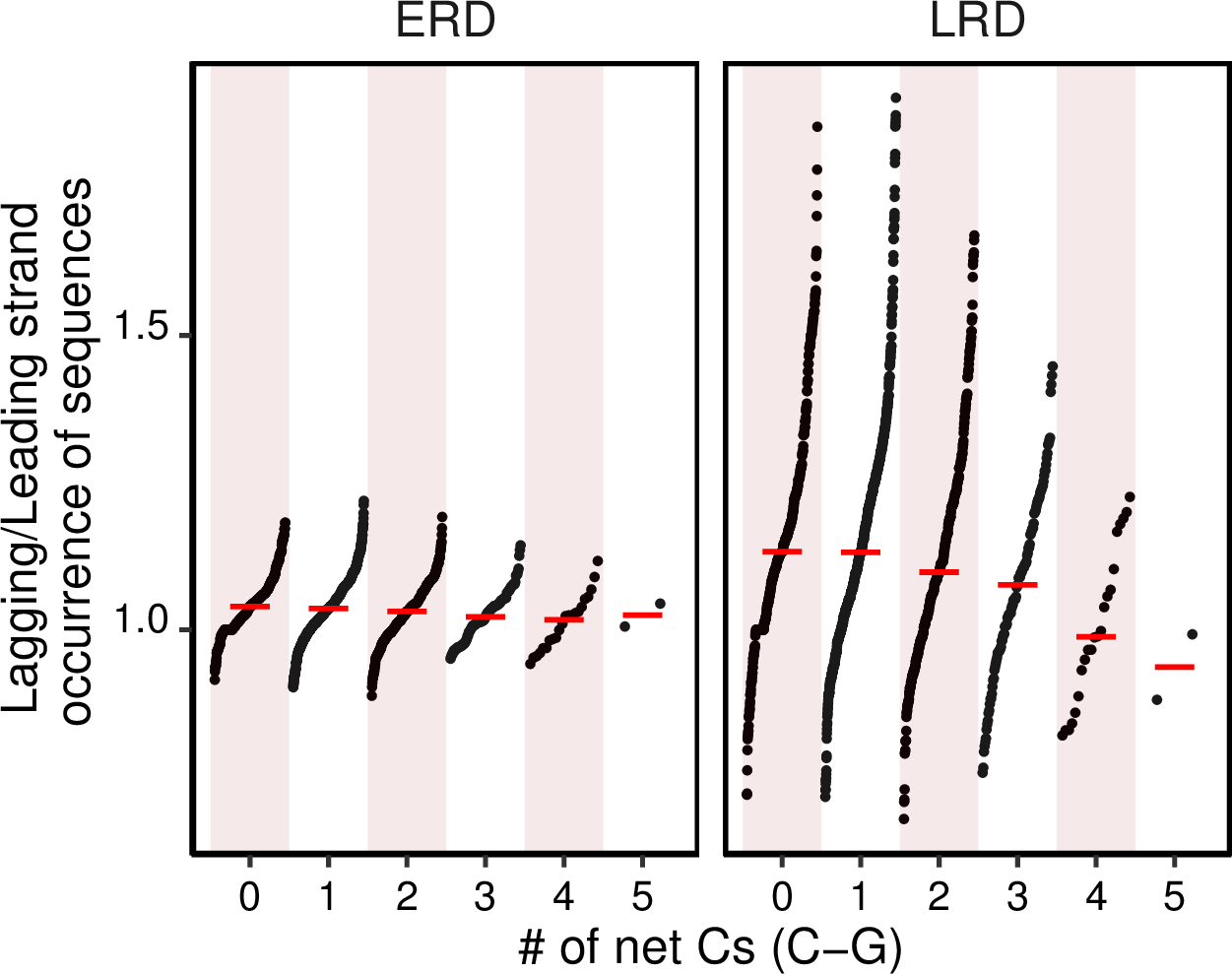

Supplement: S23 Fig — Same as Fig 4E except net Cs were calculated instead of net Ts. (TIF) [file pgen.1010426.s023.tif]

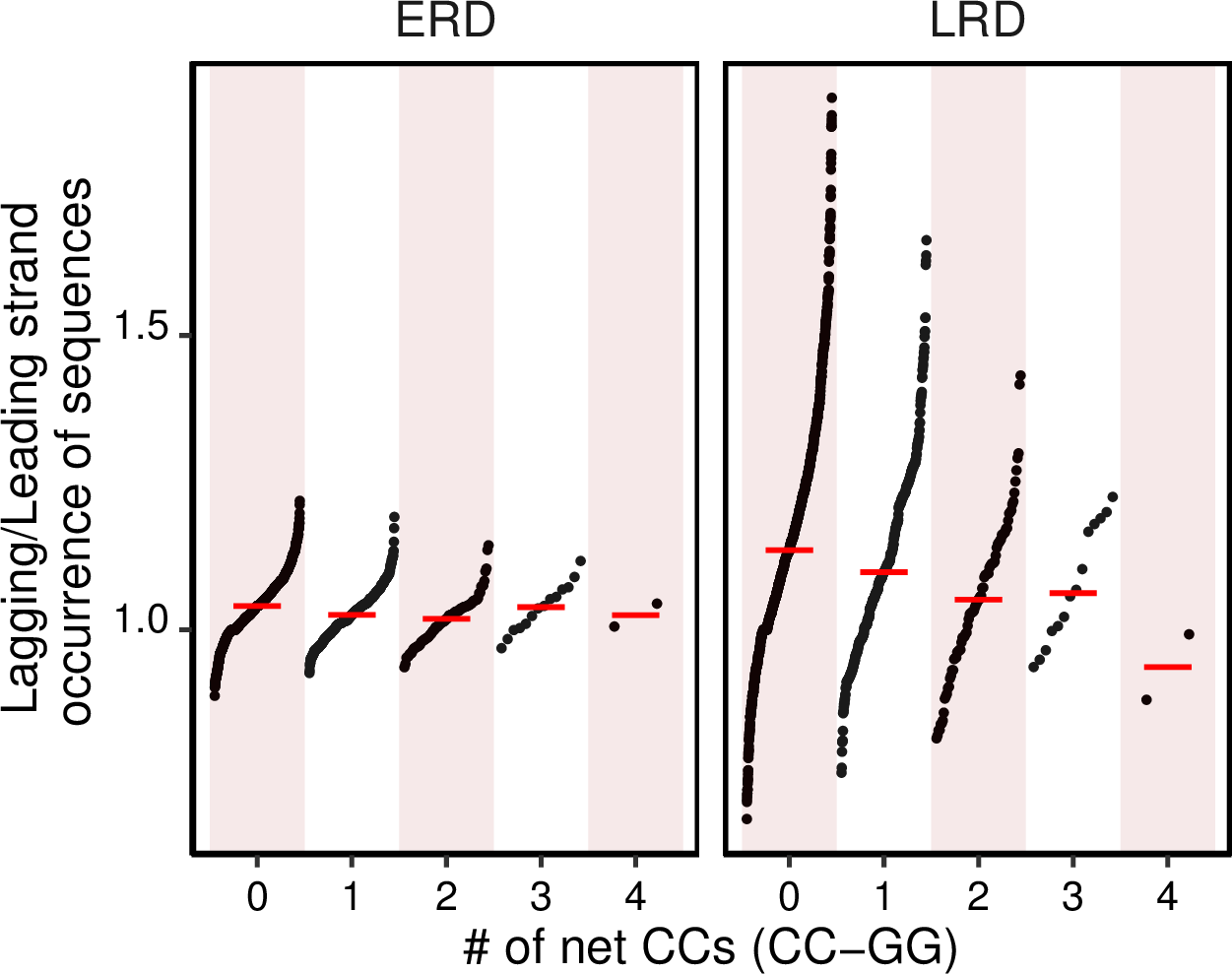

Supplement: S24 Fig — Same as Fig 4E except net CCs were calculated instead of net Ts. (TIF) [file pgen.1010426.s024.tif]

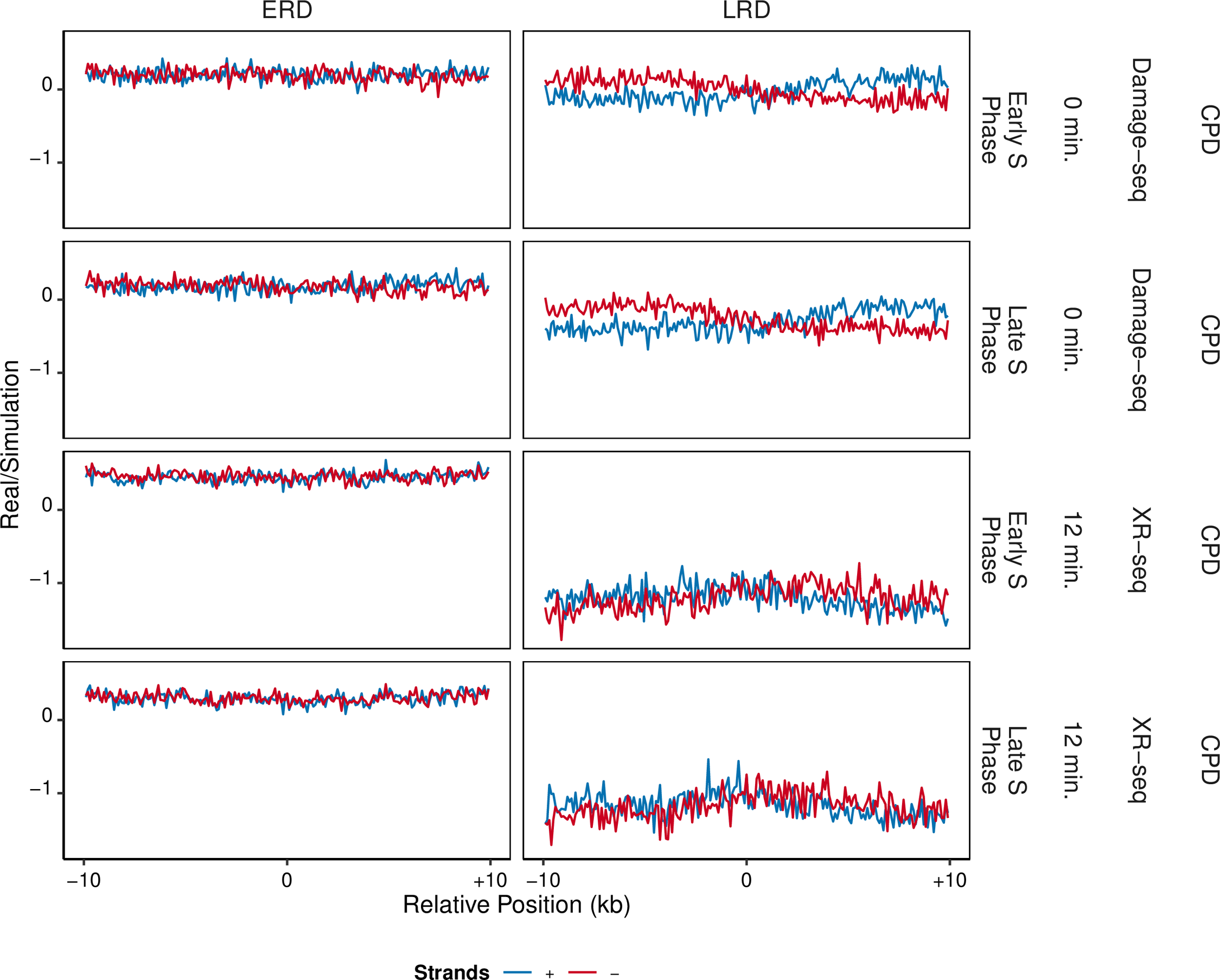

Supplement: S25 Fig — Replicate A and B are combined. (TIF) [file pgen.1010426.s025.tif]
