# Supplementary material for: Effects of replication domains on genome-wide UV-induced DNA damage and repair
Source: PLoS Genet. 2022 Sep 26;18(9):e1010426. doi: 10.1371/journal.pgen.1010426 (PMC9536635; doi:10.1371/journal.pgen.1010426)
Supplement: Attachment — Submitted filename: 22_07_17_response.docx [file pgen.1010426.s026.docx]

Response to Reviewers' Questions

We thank the Reviewers for their valuable feedback. We believe that we addressed the issues raised by the Reviewers. Here are our point-by-point responses including the major changes we incorporated in the revised version of our manuscript.

Reviewer #1: In this study, Huang et al perform genome-wide measurements of UV damage and NER activity in early S and late S phase (also EdUseq), to examine links between DNA replication program and NER. The central result of the study is summarized in Fig 2B: in late-replicating domains (LRD) there is increased repair rates in the "Late S" experiment compared to "Early S", while this is reversed in early-replicating domains (ERD), suggesting that DNA replication can locally/regionally promote activity of NER, speculatively via chromatin relaxation.

Major:
1- Repair rate is a composite of two measurements: damage and repair activity. Can they compare each of the two, between "Early S" and "Late S" experiments, in ERD and in LRD. We would need to see that it is repair (XR-Seq) that is changing not the damage. Otherwise I think the data is not compatible with the model "chromatin opens up (due to replication) to facilitate repair".

We checked Damage-seq and XR-seq separately in Fig S4, and both changed in replication phases. Notably, replication phase can affect relative DNA content in ERD and LRD, which shows up as different Damage-seq signals in early and late phases (real Damage-seq signals are normalized to simulated signals to exclude the impact of sequence context, see below for more explanation of simulation). On the other hand, as XR-seq simply captured and measured the excision products independent of damage quantification, if two regions have similar XR-seq signals while one has more damage due to more DNA content, this region should be considered to have relatively slower repair than the other one. Thus, in the main Fig 2, we only showed the “repair rate”, or “XR-seq/Damage-seq ratio” for comparison.

2- "indicating that repair rate was elevated when the region started to be replicated. This effect is likely caused by the relaxing of local chromatin during replication (33-35)." -- I think "likely" probably doesn't cut it if they wish to report this relaxing mechanism as a major finding in the Abstract or the Author Summary. Chromatin accessibility was not directly measured, unfortunately, so what remains is the proxy analysis that stratifies the data by chromatin state (Fig. 3). The results here seem unclear in terms of supporting (or not) the model. The major (by genomic coverage) active states "transcription-associated" and "low activity proximal" seem to show the same direction of effect as the major inactive states "polycomb repressed" and "HET/Rep". I am not sure this supports the chromatin opening hypothesis (the difference, though, between the active promoters and inactive promoters is notable and might support this). I think a possibly more illuminating analysis would be checking ERD vs LRD effects (like Fig 2a/2b) within each state, and/or vice versa -- stratifying for one variable while checking another.

It has been reported that replication can relax local chromatin, and repair is faster in open chromatin regions. Therefore, the raised repair in replicating regions should be, at least partially, attributed to relaxing chromatin by replication. However, we cannot exclude the contribution of other reasons. We modified the mentioned sentence.

We assume that the effects of chromatin relaxation vary among different chromatin states, so we performed the chromatin states analysis. There are two factors affecting the change of repair between early and late S phases in each state: to what extent replication relaxes local chromatin, and how the compaction of chromatin influence repair. For the latter one, we hypothesized that regions with lower repair activity should be more readily affected by chromatin relaxation. In general, the changes in the major inactive states "polycomb repressed" and "HET/Rep" are more significant, while the change in "low activity proximal" state is not significant. Although the "transcription-associated" regions are thought to be an active state, its repair activity is close to the average level of the genome. On the other hand, it is not clear to what extent replication changes the compaction of chromatin in different states in human cells. Ref. 35 reported that in yeast, genomic regions with H3K36me3 and H3K79me3, both of which are the markers of active transcription, have a slow restoration of chromatin organization after replication. In other words, replication may have a longer influence in active transcribed regions. Therefore, it is not surprising that the "transcription-associated" state has a significant change between early and late S phases, although we have no idea about the dynamics of chromatin restoration in human cells.

As Reviewer 1 suggested, we analyzed the difference between ERDs and LRDs in each state (Fig R1).

Figure R1: (A) Boxplot of normalized repair rates of CPDs at 12 minutes after UV at early and late S phases for each chromatin state. (B) Relative difference of early S phase and late S phase repair rates ([log2(RR_Early_ S Phase/RR_Late_ S Phase)] used for ERDs; [log2(RR_Late_ S Phase/RR_Early_ S Phase)] used for LRDs) for each chromatin state. The wilcoxon test was used to assess the significance of differences between ERDs and LRDs. Straight lines were ERDs, and dashed lines were LRDs. Replicate A and B are combined.

3- Regarding the strand-asymmetry in the repair process, which they ascribe to "intrinsic sequence features", here referring to extended sequence motifs of poly-T. This analysis, while interesting, seems a bit preliminary.
3a. Could they systematically check various types of sequence motifs not only polyT, to show that polyT is indeed special (if it is).

In Fig S16 we calculated the ratio of each dinucleotide on either strand and found that 1) only T-containing dinucleotides are asymmetric; 2) TT has the strongest bias in all dinucleotides; 3) the strand asymmetry in LRDs is much stronger than that in ERDs. We also analyzed the correlation of relative occurrence of net Ts on both sides of initiation zones in Fig 4E. In order to show that polyT is special, we analyzed the correlation of relative occurrence of net Ts, Cs, TTs, CCs and TCs. The results indicated that only T-containing tracks (Ts, TTs, and to a lesser extent TCs) favor the lagging strand, whereas C-containing tracks showed a rather slight decrease in lagging strands. All these trends are more obvious in LRDs. Therefore, the strong strand bias around initiation zones in LRDs is specific to polyT. We added these figures (Fig S20-S23) and revised the text.

3b. Importantly, if the sequence motif matters for the strand-asymmetry of repair rate, then this effect should be independent of the DNA replication program and should be equally observed in any location of the genome independently of the replication initiation zones.

To test whether other regions will have a similar strand difference (asymmetry in the case of initiation zones since two sides of the regions have asymmetric nucleotide distribution), we sliced the genome into 50 kb long regions and calculated the T and A percentages in those regions. T percentages provided us the potential damage sites in the forward strand, while A percentage provided the same for the reverse strand. Because sequences synthesized with lagging strand had higher T content than that of leading strand, we calculated the relative T values (T% / A%) for each region and separated regions into quartiles based on relative Ts. The quartile with the highest value tagged as “T-rich” (forward strand had similar sequence content to lagging strand) and the quartile with the lowest value tagged as “A-rich” (forward strand had similar sequence content to leading strand). Then, we mapped damage and repair signals to the selected regions and calculated the repair rate. Like the pattern of lagging strand in 4B and D, forward strand of T-rich regions had higher damage and repair than the reverse strand, but lower repair rate. Also, the forward strand of A rich regions indicated the exact opposite of that pattern. We added the figure (Fig S24) to the manuscript and revised the text.

Minor:
- Title of Fig. 1 "Ongoing replication fork stimulates nearby nucleotide excision repair" does not appear to match its contents. Fig 2 has no title.

It’s the title of the next section of the text. The actual titles of figures can be found in the section of Figure legends (p32-33).

- The dip in repair rates at the TES (Fig 1D) - is this known, if not could they comment on it?

The dip is also seen in other XR-seq experiments [e.g., Wu et al. JBC 2022, 298(5): 101863], although it is the repair rate (XR-seq/Damage-seq) here. We guess it is due to the specific sequence context and/or chromatin structure around TES, however, we didn’t perform further analysis since it is out of the scope of this manuscript.

- Also, was the repair-rate in Fig 1D around TSS/TES normalized by the simulated repair rates (controlling for sequence composition)

In Fig 1D, repair is normalized to damage, which partially excludes the impact of sequence context. We calculated the normalized version of Fig 1D and added below (Fig R2). The normalized repair rate indicates lower repair in transcribed strands and supports the conclusion that transcription-coupled nucleotide excision repair had little or no contribution to the damage repair until 12 minutes.

Figure R2: Normalized Repair rates [log2 (XR-seq_real/simulation_/Damage-seq_real/simulation_)] of template strands (TS) and non-template strands (NTS) around transcription start sites (TSS) and end sites (TES). Replicate A and B are combined.

- Reference 36 (Zheng et al.) should probably be in the introduction already. It provides important prior context for the here-reported finding of increased repair rates in early-replicating (euchromatic) domains compared with late-replicating (heterochromatic) ones.

We cited it to the introduction section (page 4 line 76, ref 28).

- Fig 2b typo "polycob"

We correct it to “Polycomb” (in Fig 3B)

- Line 178 starting with "Thus," on a first reading it was not immediately clear to me why there was a need for the additional 2h experimental timepoint. They could make their reasoning more explicit.

We advised the text.

- Lines 159-209 section was a bit technical and difficult to parse. They could consider shortening and/or rewriting for clarity.

We revised this section.

Reviewer #2: The authors attempted to build relationships between DNA damage and repair processes after UV irradiation of HeLa cells at different phases of the cell cycle and the parameters of DNA replication, including early and late replication and strand asymmetry. As shown before, early replicating regions are repaired faster than late replicating regions presumably due to open chromatin structure and active genes in those parts of the genome. They concluded that UV lesions on the lagging strand were repaired differently in late replication domains, which was probably due to an imbalanced sequence context with more T-rich sequences on lagging strands favoring cyclobutane dimer (CPD) formation. This paper contains few new observations and is presented with many conceptual and methodological problems. I also question the novelty of this paper in terms of leading versus lagging strand data when considering reference 27.

We thank the reviewer’s comment, however, there might be some misunderstandings. Our results agreed with previous findings that early replicating regions are associated with active chromatin which are repaired faster. Nevertheless, our research focused on the effects of ongoing replication on repair in local regions, and found that both ERDs and LRDs are repaired faster, especially for CPDs, when they are being replicated.

Regarding to Ref. 27, the authors took use of our published Damage-seq and XR-seq data from asynchronized NHF1 cells which are mainly in G1 phase. Therefore, whether the observed strand asymmetry is due to replication is unclear. Moreover, their analysis did not normalize repair with damage distribution, nor consider the effect of imbalanced sequence context. Indeed, their study is one of the initial motivations for us to perform this study. We conducted our experiments in synchronized HeLa cells since there are available Repli-seq and OK-seq data in this cell line. Successful cell cycle synchronization in combination with EdU-seq told us the replicating regions at time of damaging and repairing, so we can answer the question whether ongoing replication is responsible for the asymmetry between leading and lagging strands. Also, we performed simulation and normalized repair with damage distribution to exclude the influence of sequence context. Finally, we demonstrated the following conclusions. 1) The sequence context directly contributes to observed asymmetric damage (lagging strands have more CPDs) at initial time points around initiation zones in LRDs, albeit other contributor(s) also exist (Figs S7 and S8). 2) The raw repair reads have little strand asymmetry, indicating that the relative repair rate (XR-seq/Damage-seq) of lagging strands is slower than that of leading strands. This repair asymmetry would enlarge the damage asymmetry after a time course of repair, as shown in Fig S7A. We speculated that the damage asymmetry should keep increasing, then the raw repair reads would follow the trends, as shown in Ref 27 Fig 3. However, the relative repair rate of lagging strands should be always slower than that of leading strand, otherwise the strand asymmetry cannot keep increasing within 48h post UV (shown in ref 27 Fig3). 3) Most importantly, our data suggested that all these leading and lagging strand asymmetries are mostly, if not completely, independent of ongoing replication. In a sense, this is a negative result, however, we believe it is the right answer to the initial question, and is valuable to this field.

1) They used two methods to assess DNA damage: damage-seq and XR-seq. XR-seq measures a combination of initial damage levels and repair efficiency in the form of in vivo excised short oligonucleotide fragments that are sequenced. Because these are two very different methods, it seems questionable to specifically infer DNA repair rates by normalizing XR-seq versus damage-seq data. A more straightforward approach would be to perform damage-seq at various time points.

2) When looking at XR-seq data from a 12-minute time point after irradiation, this is likely measuring only very fast repair kinetics and should reflect primarily (6-4) photolesions. The authors are not measuring real repair rates with XR-seq other than a snapshot of repair, and only two timepoints were used (12 min and 2 hours).

Response to points 1 and 2: XR-seq is a method to directly measure NER independent of damage detection, so it can detect very weak repair when the damage distribution just changes a little. The distribution of excision products, as the reviewer mentioned, depends on both repair capacity and damage distribution, so we need to normalize XR-seq with Damage-seq. They are quite different methods, but they have some common properties making them comparable. Firstly, they are both strand-specific and reach base resolution (for XR-seq, the dual incision sites are determined at single-nucleotide resolution, while the possible damage sites are near-nucleotide resolution, which is enough for comparing with Damage-seq in this manuscript). Secondly, they use the same damage-IP strategy and damage antibodies for IP, so normalizing XR-seq with Damage-seq can eliminate any possible bias caused by the damage IP step.

Obtaining a snapshot can be considered as a weak point of XR-seq in some cases, but is also an advantage in other cases. In this study, we wanted to assess the impact of ongoing DNA replication on local repair at genomic level. Of note, replication is a dynamic process which takes ~6-8h in HeLa cells. On the other hand, measuring accumulated repair by subtraction of damage usually requires hours to see obvious changes in damage distribution. During this process, the replication forks are moving, and the replicating regions are changing. Therefore, measuring snapshots of repair by XR-seq is ideal.

It is well known that DNA replication would slow down or pause upon damaging, which may complicate our analysis. Thus, we decided to measure repair at a very early time point (12 min) before the disrupted replication exerts its impact. As the reviewer said, (6-4)PP is the major repair products at that time point, however, CPD is also being repaired with a lower rate. In XR-seq, repair of both lesions is separated by IP with antibodies specific for the different lesions. According to the reviewer’s suggestion, we also calculated accumulated repair of CPD by subtracting damage at 2h from initial damage (0h). Indeed, accumulated repair results agreed with the normalized repair rate in Figure 4D, which indicated more efficient repair of leading strands. We added the figure (Fig S12) to the manuscript and revised the text.

3) Figure 1D:
The strong DNA repair peak at the TSS contrasts entirely with a dominant mutation peak in melanoma at the TSS as published by Perera et al (Nature, 2016).

In this study, we did not focus on the details of repair around TSSs. Indeed, Perera et al. and Sabarinathan et al. identified a dip of repair close to TSSs from our published XR-seq data by analyzing repair over a small scale (hundreds of base pairs), which corresponds to a mutation peak. XR-seq of HeLa cells from our recent paper [Wu et al. JBC 2022, 298(5): 101863] also showed a dip near TSSs when repair was calculated at single base resolution.

To check the dip of repair with our samples, we plotted the RPKM values of damage and repair for both (6-4)PP and CPD samples at 12 minutes (Fig R3). As expected, repair values of both samples had a dip close to TSS, however low numbers of damage at the same location prevented us from observing the dip in the repair rate calculation. In addition, when we plotted the simulation-normalized repair rates around TSS, the dip became visible for CPD samples (Fig R2).

Figure R3: RPKM values of damage and repair events on template strands (TS) and non-template strands (NTS) around transcription start sites (TSS) and end sites (TES). Replicate A and B are combined.

4) The authors repeatedly work with simulated damage and repair rates but never explain how they measured these. What is the purpose of using “simulated” data rather than real data?

Damage and repair reads were simulated by boquila (https://github.com/compGenomeLab/boquila) tool. boquila calculates the overall frequency of nucleotides among the given reads and choses the regions with the most similar sequence distribution as synthetic reads. The main idea behind using simulated damage and repair reads in comparison to the real reads is to eliminate any possible bias caused by the sequence distribution. For example, a region with a high CPD damage may harbor those damages simply due to having more TT dipyrimidines than other genomic regions. If this is the case, simulated reads will control for damage levels at the same region and normalizing real reads with the simulation would flatten this high damage profile.

5) On line 184, the authors state that transcription-coupled repair might play a role at 2h but not at 12 min. That makes little sense to me.

In this section, we wanted to investigate the strand asymmetry of NER between leading and lagging strands. However, it is well known that the transcription-coupled nucleotide excision repair (TCR) sub-pathway has very strong strand asymmetry in genic regions, and it plays an important role in the repair of CPDs. As shown in Fig 1D, TCR seems to have little contribution to CPD repair at 12 min. Although we don’t know the actual reason, it means we don’t need to consider the impact of TCR on strand asymmetry at 12 min. In contrast, Fig S3D indicated that there was a strong strand bias due to TCR at 2h. Fig S14A showed that the directions of genes around initiation zones are asymmetric. Thus, the effect of TCR on the strand asymmetry around initiation zones needs to be considered. This explains why we performed the analysis in Figs S13 and S14B-C after removing transcribed regions.

6) Some sections of the paper are confusing. See lines 188/189 and lines 206-208. Part of the ambiguity is probably caused by the conflation of DNA repair and DNA damage levels within XR-seq data.

We revised these sentences for clarification.

7) Figure 4:
The repair data for panels B and C look different even though they both are from the same time point.
What do the authors mean by left replicating and right-replicating?
Panel E. The number of dipyrimidines is more important than the net numbers of T-A.

Panel B showed the real/simulated damage/repair separately and found that they all had strand asymmetry, to different extent. That means the sequence context had a direct influence on both damage and repair. Moreover, repair measured by XR-seq are determined by both damage distribution and repair capacity. Therefore, in panel C, we compared the repair rate while controlling for the sequence context and damage distribution by normalization. So Panels B and C do look different.

Because both replication forks from a specific replication origin move bidirectionally, we tagged one direction as left-replicating (plus strand being leading strand), while the other direction as right-replicating (plus strand being lagging strand).

Firstly, as the reviewer said, CPDs are formed not only on TT, but also on TC, CT and CC (CC is very rare). Secondly, what we showed in panel E is the ratio of CPD on lagging and leading strands. That means we need to consider the chance of CPD formation on both strands. For instance, the sequence “TTAA” and “TTGG” both have one “TT”, so they should have a similar chance to form a TT-CPD. However, the opposite strand of “TTAA” but not “TTGG” also has a “TT”, so the strand asymmetry of CPD is different for the two sequences. Thus, the difference of T-containing dipyrimidines on two strands, or the net number of T-containing dipyrimidines might be a better indicator. Here we used net T or “T-A” to simplify our analysis, as the net T should be positively correlated to net number of T-containing dipyrimidines. In addition, we analyzed the correlation of relative occurrence of net TTs and TCs on both sides of initiation zones, and found that TTs, and to a lesser extent TCs, showed a similar pattern as Ts which favor lagging strands.

8) The authors used replication data from HeLa cells and from two fibroblast cell lines for comparisons with mutation data from melanomas. However, how much are the replication patterns cell type-dependent? According to Figure 5, the number of shared initiation zones is quite limited between the three cell lines, and HeLa is the greatest outlier. For these reasons it seems inappropriate to extrapolate from these cell line replication data to mutations in melanocytic tumors.

The replication timing (RT) is determined by the order of replication origin firing, which is correlated with high-order chromatin structure, namely topologically associated domains, and would change when cell fate alters. Nonetheless, as discussed in ref. 22, RT constitutive domains exist, and about half of replication domains switch replication timing during normal development and progression of disease including cancer. Therefore, due to the limited availability of RT data, using RT data from different cell lines is acceptable. For instance, ref. 21 used RT data from six lymphoblastoid cell lines to compare with mutation landscapes in different cancers. Another example is ref. 23 which used RT data from somatic cell lines to compare with germline mutations. Initiation zone is a conception that is tightly related to replication timing. Indeed, initiation zones are determined by the switch of replication direction, which can be measured by OK-seq as we did, or by replication timing (Repli-seq) as in refs. 21 and 25. Since OK-seq and Repli-seq data for melanoma are not available, we could only use data from other cell lines. In order to reduce the impact of different cell lines, we used overlapped initiation zones of three cell lines based on the hypothesis that the common initiation zones among all three distinct cell lines should be the constitutive ones. Our analysis indicated that the overlapping initiation zones are more robust (higher score in Fig 5B), and initiation zones with higher score have stronger strand asymmetry of mutation in late replication domains. These results indicated that the strand asymmetry of mutations around initiation zones in LRDs is true. Indeed, as claimed in ref. 23, “The correlation reported herein should therefore be regarded as a lower limit estimate of actual dependence of mutation rate on replication timing”, and the actual strand asymmetry of mutation around initiation zones in LRDs would be even stronger if using actual replication data from melanoma.

9) They used non-physiological UVC radiation. Most comparisons from such data cannot be related to human skin cancer mutations.

The physiological UV irradiation, or UV in solar irradiation, is the mixture of UCB and UVA. Although UVA can induce oxidative DNA damage, the main mutagenic DNA damage caused by solar irradiation is CPDs and (6-4)PPs, which is also induced by UVC, albeit the ratio of induced (6-4)PP and CPD is different. Since using UVC produces the mutagenic CPD and (6-4)PP damage without the confounding oxidative DNA damage generated when using simulated or real solar irradiation, it is widely accepted to use UVC in the study of NER. Indeed, our XR-seq data from UVC-irradiated cells were demonstrated to be well correlated to mutation landscapes in human skin cancers by other groups (refs. 15 and 16).

10) Methods: What does it mean that replicates A and B were combined throughout the analysis?

Initially, we checked the correlation between the replicates and found that they were highly correlated. Therefore, we combined the pre-processed bed files of replicates to increase the sequencing depth.

Abstract: It is speculative to say that replication contributes to accumulating T-tracts and high AT content on lagging strands of initiation zones during evolution. There is no data to support such an assumption.

We appreciate the reviewer’s comment and modified this sentence in “AUTHOR SUMMARY” for clarification.

Reviewer #3: In “The effects of replication domains on the genome-wide UV-induced DNA damage and repair,” Huang et al., investigate the impact of DNA replication of UV damage repair efficiency. It was previously known that UV-induced mutations in cancers are increased while nucleotide excision repair (NER) is decreased in late replicating, lowly expressed heterochromatic domains of the human genome. However, the relative contribution of transcription and replication to opening chromatin to allow NER to function in heterochromatic domains is unknown. The authors therefore utilize Edu-seq to map early and late replicating domains in HeLa cells and then independently map CPDs and 6-4 photoproducts using damage-seq and XR-seq methods in synchronized cells in either the early or late phase of DNA replication. The authors find that NER efficiency in both the early and late replicating domains is elevated when each domain is being actively replicated, suggesting that DNA replication plays some role in providing access of NER to UV induced lesions. This effect is most dramatic for CPDs over 6-4 photoproducts. Effects of active replication on NER repair occur in multiple chromatin domains across these regions. Damage-seq, XR-seq reads, and UV signature mutations in melanoma also occurred more readily in replication initiation zones on the lagging strand, consistent with previous results (2). However, T:A richness of the lagging strand was identified and would provide more potential sites of UV lesions to be formed on the lagging strand, suggesting some of the lagging strand bias may be due to sequence context. Overall, the manuscript is fairly well written and easy to follow. The determination that replication increases NER efficiency is a significant discovery towards our understanding of how mutational heterogeneity is established in human cancer cells. I believe addressing the following specific points will strengthen the manuscript.
Specific points
1. In line 113, the authors use the lack of transcriptional strand bias at 12 min post-exposure to argue that their analysis on replication does not have the exclude the impacts of transcription. However, transcription is still proceeding during these 12 minutes and can also impact global genomic NER, especially in heterochromatic regions (3). Because of the strong association of replication timing with transcriptional activity, it is difficult to separate the contribution of each of these processes on chromatin state through mapping techniques. Additional discussion of this point or measuring damage-seq and XR-seq in the presence of a transcription inhibitor like actinomycin D would solidify this statement.

We agree that transcribed regions also have higher GGR which was reported by us and other groups, but the contributions of chromatin structure at transcribed regions and transcription itself cannot be separated. Indeed, both chromatin structure and transcription might affect GGR by changing the accessibility to repair factors. We speculated that replication could affect GGR but not TCR also through altering accessibility, so we had the plan to exclude TCR by choosing intergenic regions, as we did in Figs S13 and S14 for the 2h time point. Since there was no obvious TCR at 12 min, we did not need to do that for the 12 min time point. Although transcription would also affect GGR, we think it is not necessary to use a transcription inhibitor to exclude the impact of transcription on GGR for two reasons. Firstly, we already performed cell cycle synchronization and UV irradiation, and it is unclear whether additional transcription inhibition has any unexpectable effects. More importantly, we always compared repair rates in the same regions between different cell cycle phases. Only a small portion of genes have different transcription levels between early and late S phases [Yildirim et al. Cell Rep. 2020;31(6):107629.], so the effect of transcription on GGR should be largely unchanged for the same region. As shown in Figs 2 and 3, ERDs and active chromatin regions are repaired faster than LRDs and inactive regions, and transcription should contribute to this pattern. Nevertheless, most regions are repaired faster when being replicated, regardless of their original repair rates and transcription activities.

2. The effect size of DNA replication on promoting NER appears to be relatively small. In Fig. 2B its clear that despite ongoing replication, NER in late replicating regions is still greatly inhibited compared to in early replicating regions. Is this difference do to the higher transcriptional activity of the early replicating domains? Stating specific fold changes or odds-ratios throughout the text would help establish how much of an impact replication is having on NER rate as would a discussion of how these effect sizes compare to other influences on NER.

We appreciate the reviewer’s suggestion. Regarding a specific region of the genome, replication is a transient process, thus its impact is quite limited in both time and space. Due to the limitations of the technique, we could only study the effect of replication on repair at a replication domain-scale, so the observed effect of replication on repair is lower than that of chromatin compaction and transcription activity. In other words, the genome-wide repair profile mainly depends on chromatin compaction and transcription activity at a replication domain-scale, while replication just exerts an additional impact on it. As an example, we plotted chromosome-wide repair rate as well as fold change of repair between early and late S phases at 12 min post UV in chromosomes 1 and 19 (Fig S5) and added it to the manuscript.

3. In line 148, the authors state that the difference between repair efficiency with and without replication is minor for more efficiently repaired regions. However, in fig. 3 they show that inactive promoters, CTCF insulators, and Low activity proximal states are three of the four non-significant chromatin states and they have middle to low repair rates indicated in Fig. 3A. This seems to be an over-simplification of the data.

As discussed in the response to Reviewer 1, there are two factors affecting the change of repair between early and late phases in each chromatin state: to what extent replication relaxes local chromatin, and how the change of chromatin compaction influences repair. We speculated that the latter one is minor for more efficiently repaired regions; however, the contribution of the first factor is unclear. Moreover, the significance also depends on additional factors such as sample size. We added the number of each state in ERDs and LRDs to Figs 3 and S6, and revised the manuscript for clarification.

4. The authors interpret the lagging strand bias of UV damage, NER, and mutation to be caused by an underlying sequence bias for T in the lagging strand which would provide more dipyridines for damage on the lagging strand. However, the biases seen for UV damage and NER is greater than the simulated bias based on sequence content alone. Additionally, the mutation analysis in Fig. 5 standardizes by sequence content. This suggests that other factors also influence the increased lesions on the lagging strand. What some of these possibilities are should be discussed.

We agree that the strand asymmetry is different from simulation that real damage has a stronger bias than simulation while real repair has a weaker bias than simulation, so there must be other factors that we have not considered in the simulation. However, observing the same pattern in both early and late S phases excludes the possibility that it is due to replication. To be honest, we don’t know the exact mechanism. Nonetheless, we have some ideas. For damage formation, CPDs are likely to be induced at three or more continuous Ts, which are specifically enriched on the lagging strand around initiation zones in LRDs. This pattern is not reflected in our simulation. In addition, the enriched T-tracks might form more than one CPD close to each other which could interfere with repair, although the chances of forming clustered lesions is low.

5. The use of OK-seq defined initiation zones seems to limit the focus of the analysis to specific regions of the genome. Are similar effects seen if overall replication direction assessments (like those is (1), are used?

We appreciate the reviewer’s question. To address this question, we plotted chromosome-wide repair asymmetry along with replication timing in chromosomes 1 and 19. Although some of the one-direction replicating regions show the same trend of repair strand asymmetry, no general conclusions can be drawn. It is not surprising, since the pattern around initiation zones we observed is due to the sequence context rather than replication. As discussed in the response to Reviewer 1, we could see similar repair asymmetry in regions with A/T bias. Therefore, the repair asymmetry is not directly related to replication direction. We added this figure (Fig S5) to the manuscript.

6. While generally well-written, there are small typos throughout the manuscript that should be corrected. An example is in the title, which should probably be: The effects of replication domains on genome-wide UV-induced DNA damage and repair. The inclusion of the word “the” doesn’t make sense.

We thank the reviewer’s suggestion and modified our title.

References:
1. Haradhvala NJ, Polak P, Stojanov P, Covington KR, Shinbrot E, et al. 2016. Mutational Strand Asymmetries in Cancer Genomes Reveal Mechanisms of DNA Damage and Repair. Cell 164:538-49
2. Seplyarskiy VB, Akkuratov EE, Akkuratova N, Andrianova MA, Nikolaev SI, et al. 2019. Error-prone bypass of DNA lesions during lagging-strand replication is a common source of germline and cancer mutations. Nat Genet 51:36-41
3. Zheng CL, Wang NJ, Chung J, Moslehi H, Sanborn JZ, et al. 2014. Transcription restores DNA repair to heterochromatin, determining regional mutation rates in cancer genomes. Cell Rep 9:1228-34
