# Supplementary material for: Effects of replication domains on genome-wide UV-induced DNA damage and repair
Source: PLoS Genet. 2022 Sep 26;18(9):e1010426. doi: 10.1371/journal.pgen.1010426 (PMC9536635; doi:10.1371/journal.pgen.1010426)
Supplement: Attachment — Submitted filename: 22_09_05_response.docx [file pgen.1010426.s027.docx]

Response to Reviewers' Questions

We thank the Reviewers and the Editor for their valuable feedback. We fixed seed number for random processes in the algorithms for reproducibility, therefore, figures 1-5 and S3-25 were reproduced. In addition, we added picard toolkit to the reference list (ref. 54). Here are our point-by-point responses.

Reviewer #1: In the revised manuscript, Huang et al performed some additional analysis in response to my queries. I have only minor suggestions remaining. Generally I think that the study and presentation has been improved by the revisions.

There is added value in the analyses that clarified the role of various types/lengths of repeats. They should consider plotting some of this data as an additional main figure rather than keeping it buried in Fig S20-24.

We appreciate the reviewer’s suggestion. Fig S20 and S24 are imbedded into main Fig 4 (Fig 4F-G).

Regarding the role of chromatin opening mediating the mechanism they report, we now have it clear in the text their study does not in fact have data to support this "This effect could be, at least partially, attributed to the relaxing of local chromatin during replication (34-36)." I think they should also correct the Abstract by removing "by relaxing surrounding chromatin".

The mentioned sentences are removed.

I think their Fig R1A is an interesting visualization because it supports that the more likely causal role in shaping repair rates lies with replication time, rather than with eu/heterochromatin, if I am reading this correctly. I would suggest to include this figure in the manuscript as it provides a different, more informative emphasis than Fig 3 currently provides.

Fig R1A and corresponding text are added to the manuscript (Fig S7).

Reviewer #2: I am overall satisfied with the authors' responses.

Reviewer #3: Dr. Ogun Adebali and colleagues have submitted their revision of “Effects of replication domains on genome-wide UV-induced DNA damage and repair.” In general, the authors have addressed my previous comments. However, I have some minor suggestions for clarification to the text.

1) When addressing the differential impact of replication to chromatin compaction on NER repair rates, the authors conclude in the rebuttal that “genome-wide repair profile mainly depends on chromatin compaction and transcription activity, while replication exerts an additional impact.” I did not see this statement added to the manuscript. I think it should be included somewhere.

We appreciate the reviewer’s suggestion. This statement is added to the last paragraph of Discussion.

2) The lack of significance for differences in repair efficiency while replicating or not in inactive promoters and CTCF insulations are clearly due to low numbers, which the authors now provide. This leaves only Low activity proximal sites as non-significant. However, the numbers provided suggest that repressed polycomb and heterochromatin are significant, but in the opposite direction. This would fit the authors’ interpretation. It may be helpful to provide an odds-ratio to help emphasize this, instead of just providing the statistical significance.

For both polycomb and heterochromatin regions, ERDs are repaired faster in early replication phases and LRDs are repaired faster in late replication phases, which fits our interpretation, as the reviewer mentioned. We calculated the odds-ratios between early and late replication phases as the reviewer suggested and added the figure to the manuscript (Fig S8).

3) I am unclear how Figure S5 contains information about repair strand symmetry. Please clarify this.

The reviewer has a valid point here. Fig S5 does not contain any information regarding the repair strand symmetry. We corrected this mistake which was overlooked during the transition across revised manuscript versions.

Have all data underlying the figures and results presented in the manuscript been provided?

Large-scale datasets should be made available via a public repository as described in the PLOS Genetics data availability policy, and numerical data that underlies graphs or summary statistics should be provided in spreadsheet form as supporting information.

Reviewer #1: Yes

Reviewer #2: Yes

Reviewer #3: No: There does not appear to be an numerical data for graphs or summary statistics in spreadsheet form.

We added a compressed file containing data frames of each graph in csv format.
